# Enhancement of drug delivery through fibroblast activation protein–targeted near-infrared photoimmunotherapy

**DOI:** 10.1172/jci.insight.195776

**Published:** 2025-12-22

**Authors:** Seitaro Nishimura, Kazuhiro Noma, Tasuku Matsumoto, Yasushige Takeda, Tatsuya Takahashi, Hijiri Matsumoto, Kento Kawasaki, Hotaka Kawai, Tomoyoshi Kunitomo, Masaaki Akai, Teruki Kobayashi, Noriyuki Nishiwaki, Hajime Kashima, Takuya Kato, Satoru Kikuchi, Shunsuke Tanabe, Toshiaki Ohara, Hiroshi Tazawa, Yasuhiro Shirakawa, Peter L. Choyke, Hisataka Kobayashi, Toshiyoshi Fujiwara

**Affiliations:** 1 Department of Gastroenterological Surgery,; 2 Department of Oral Pathology and Medicine, and; 3 Department of Pathology and Experimental Medicine, Okayama University Graduate School of Medicine, Dentistry, and Pharmaceutical Science, Okayama, Japan.; 4 Center for Innovative Clinical Medicine, Okayama University Hospital, Okayama, Japan.; 5 Department of Gastroenterological Surgery, Hiroshima City Hiroshima Citizens Hospital, Hiroshima, Japan.; 6 Molecular Imaging Branch, Center for Cancer Research, National Cancer Institute, NIH, Bethesda, Maryland, USA.

**Keywords:** Gastroenterology, Oncology, Extracellular matrix

## Abstract

The tumor microenvironment plays a key role in cancer progression and therapy resistance, with cancer-associated fibroblasts (CAFs) contributing to desmoplasia, extracellular matrix (ECM) remodeling, and elevated interstitial fluid pressure, all of which hinder drug delivery. We investigated fibroblast activation protein–targeted (FAP-targeted) near-infrared photoimmunotherapy (NIR-PIT) as a strategy to improve drug penetration in CAF-rich tumors. In clinical esophageal cancer samples, FAP expression strongly correlated with increased collagen I, hyaluronic acid, and microvascular collapse. CAF-rich 3D spheroids demonstrated elevated ECM deposition and significantly impaired drug uptake compared with CAF-poor models. FAP-targeted NIR-PIT selectively reduced CAFs, reduced ECM components, and restored drug permeability. In vivo, FAP-targeted NIR-PIT enhanced the accumulation of panitumumab and Abraxane in CAF-rich tumors and improved antitumor efficacy when combined with chemotherapy. These findings highlight FAP-targeted NIR-PIT as a promising therapeutic approach to remodel the tumor stroma and overcome drug resistance in desmoplastic solid tumors.

## Abstract 2

Photoimmunotherapy targeting fibroblast activation protein reduces cancer-associated fibroblasts and improves drug delivery in tumors with abundant stroma and fibroblast activation

## Introduction

The tumor microenvironment (TME) plays a crucial role in supporting cancer progression and contributing to therapy resistance (1, 2). Chemoresistance can arise from intrinsic changes in cancer cells and from impaired drug delivery due to structural and functional barriers within the TME (3, 4). Among the key TME components, cancer-associated fibroblasts (CAFs) are believed to play a central role in both tumor growth and therapeutic resistance (5, 6).

Esophageal cancer, a representative solid malignancy, has a poor prognosis, with a 5-year survival rate below 20% (7–9). Although treatment options, such as immune checkpoint inhibitors, have expanded in recent years, overall survival (OS) in patients with recurrent or metastatic esophageal cancer remains limited; typically the median OS is approximately 1 year. One major contributing factor to this poor outcome is chemoresistance (10–12).

CAFs have been associated with poor prognosis in esophageal cancer and are known to contribute to drug resistance (13–15). Similarly, in other solid tumors, CAFs contribute to drug resistance through mechanisms like the secretion of stromal cell–derived factor-1, which promotes gemcitabine resistance in pancreatic cancer (16), and IL-6, which facilitates epithelial-mesenchymal transition and drug resistance in non–small cell lung cancer (17). CAFs also play a significant role in extracellular matrix (ECM) production and remodeling, contributing to desmoplasia, a condition characterized by excessive stromal proliferation. The desmoplastic reaction can establish physical barriers that hinder drug delivery and contribute to increased interstitial fluid pressure due to tumor stiffness. This phenomenon may lead to vascular compromise and impaired efficacy of drug administration (18–21).

Therapies targeting CAFs may offer a potential strategy for overcoming fibroblast-associated chemoresistance; however, CAF-targeted therapies have not been widely adopted in clinical practice. Challenges include the lack of CAF-specific markers and the heterogeneity of CAFs, including both tumor-promoting and tumor-suppressing subpopulations (6, 22). One promising CAF marker is fibroblast activation protein (FAP), a membrane-bound serine protease highly expressed in CAFs. Although FAP-targeted therapies have been explored, significant adverse effects and phase II trial failures have hindered their development (23–25). To address these challenges, we developed FAP-targeted near-infrared photoimmunotherapy (NIR-PIT). NIR-PIT is a specific therapy that rapidly induces necrosis of targeted cells (26). Moreover, as a localized treatment, it is expected to have fewer systemic side effects compared with the adverse events observed in previous clinical trials of systemic CAF-targeted therapies.

An additional and unique feature of NIR-PIT is its ability to markedly enhance drug delivery through the super-enhanced permeability and retention (SUPR) effect (27, 28). This phenomenon represents an augmentation of the classical enhanced permeability and retention (EPR) effect, whereby nanosized drugs preferentially accumulate in tumors owing to their leaky vasculature and poor lymphatic drainage (28). Following NIR-PIT, rapid perivascular cancer cell death and vascular dilation further increase intratumoral permeability, leading to considerably greater drug accumulation. Traditionally, this effect has been demonstrated with nanosized particles (“nano-SUPR”), but a recent study has demonstrated that even micron-sized particles and ultrasound microbubbles can accumulate in tumors after NIR-PIT (“micro-SUPR”) (29). Moreover, contrast-enhanced ultrasound imaging with Sonazoid-derived microbubbles has been shown to correlate with therapeutic effects, suggesting a feasible and noninvasive approach for the clinical monitoring of the SUPR effect (29).

For FAP-targeted NIR-PIT, we used sibrotuzumab, a humanized monoclonal antibody that has been in early clinical trials and was conjugated to IR700 (24). The CAF-targeted NIR-PIT we developed, which specifically targets FAP, initially demonstrated antitumor efficacy in CAF-rich tumors (30). Subsequently, we established the effectiveness of dual-targeted NIR-PIT by combining FAP-targeted NIR-PIT with EGFR-targeted NIR-PIT (31). Furthermore, FAP-targeted NIR-PIT using sibrotuzumab exhibited similar therapeutic effects (32). Additionally, this approach has been shown to activate host immunity (33).

**Figure 1:**
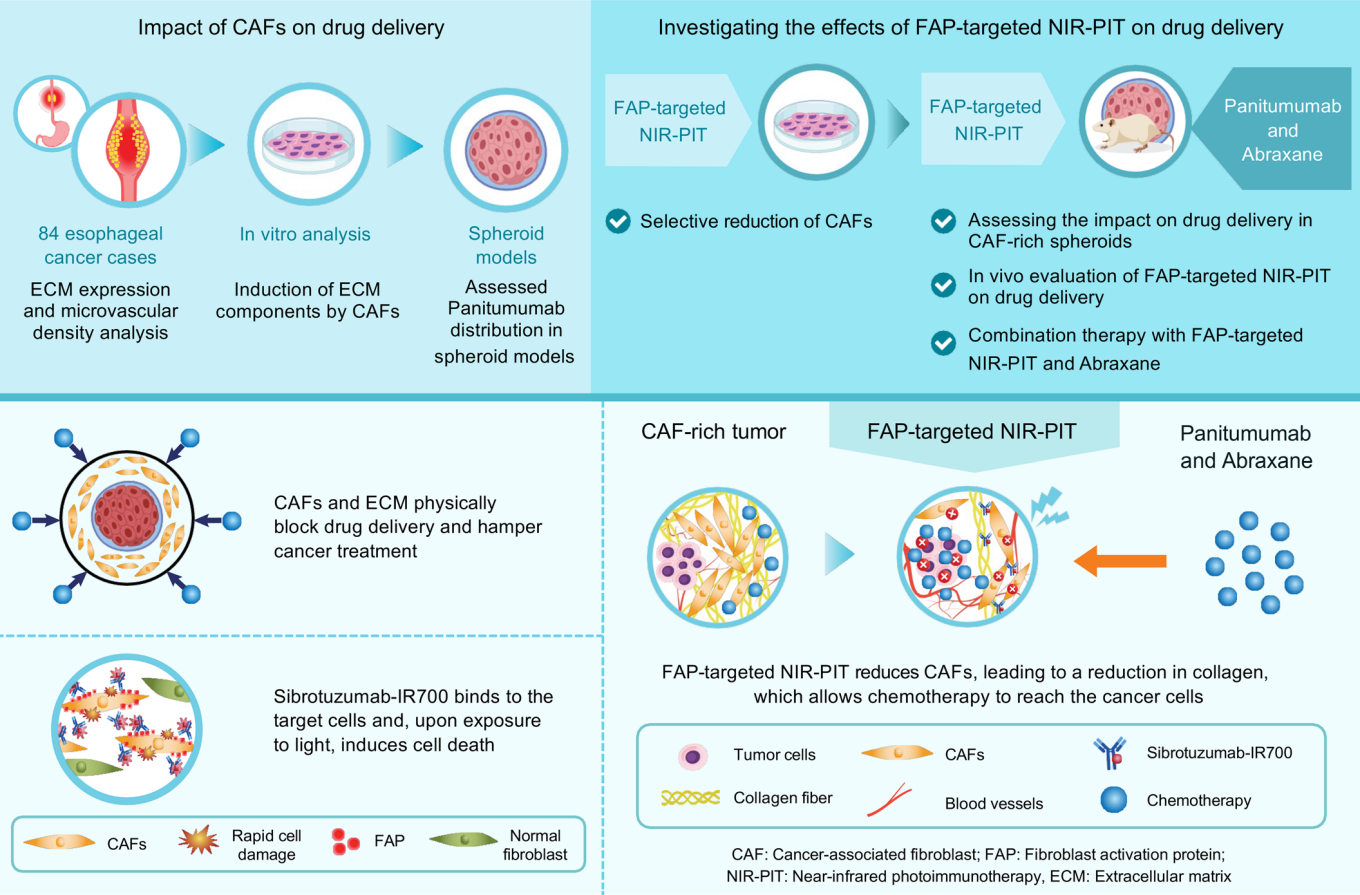
Overview of the experimental workflow and key findings of FAP-targeted NIR-PIT. Top: Experimental strategy to investigate the role of CAFs in drug delivery and the therapeutic potential of FAP-targeted NIR-PIT. The study included ECM and microvascular density analysis in 84 esophageal cancer cases, in vitro assessment of ECM induction by CAFs, and drug distribution analysis in 3D spheroid models. The effects of FAP-targeted NIR-PIT were further examined through selective reduction of CAFs, spheroid drug uptake analysis, in vivo tumor drug accumulation, and combination therapy with panitumumab and Abraxane. Bottom: Summary of key findings. CAF-rich tumors exhibited ECM accumulation and impaired drug delivery. FAP-targeted NIR-PIT selectively reduced CAFs, reduced collagen content, and improved drug penetration, thereby enhancing the intratumoral chemotherapeutic agent accumulation.

In this study, we hypothesized that FAP-targeted NIR-PIT would induce the SUPR effect, as previously reported in NIR-PIT studies targeting cancer cell–surface antigens (27), thereby improving drug delivery by reducing intratumoral pressure and enhancing vascular permeability. We also propose that targeting FAP-positive CAFs may induce ECM remodeling, potentially further reducing physical barriers and facilitating the distribution of therapeutic agents within tumors. We believe the combination of these mechanisms — the SUPR effect and ECM remodeling through CAF reduction — could improve therapeutic outcomes. To test this hypothesis, we investigated whether CAFs contribute to increased stromal pressure and impaired drug delivery, and whether FAP-targeted NIR-PIT can enhance drug delivery in CAF-rich tumors, improving antitumor effects. Figure 1 summarizes the overall study design and key findings.

## Results

### ECM and microvascular density associated with the prognosis of esophageal cancer.

Based on previous studies (21), we hypothesized that increased stromal volume elevates intratumoral pressure, leading to vascular collapse and reduced drug delivery. We focused on the ECM and vascular diameters. For the ECM, we focused on its main components: collagen and hyaluronic acid (HA) (34–36). Collagen contributes to blood vessel compression and hinders drug delivery (37, 38). HA also plays a significant role in vascular collapse and inhibition of drug penetration (36, 39). For vascular evaluation, we measured microvascular density (MVD) and the maximum short diameter (36). To explore the relationship between ECM expression and clinical outcomes in esophageal cancer, we assessed the expression levels of collagen I (Col I), the predominant form of collagen, and HA in resected tumors using IHC. Initially, 149 patients who underwent esophagectomy were included; after exclusion criteria were applied, 84 cases were selected for analysis (Supplemental Figure 1A; supplemental material available online with this article; https://doi.org/10.1172/jci.insight.195776DS1).

**Figure 2:**
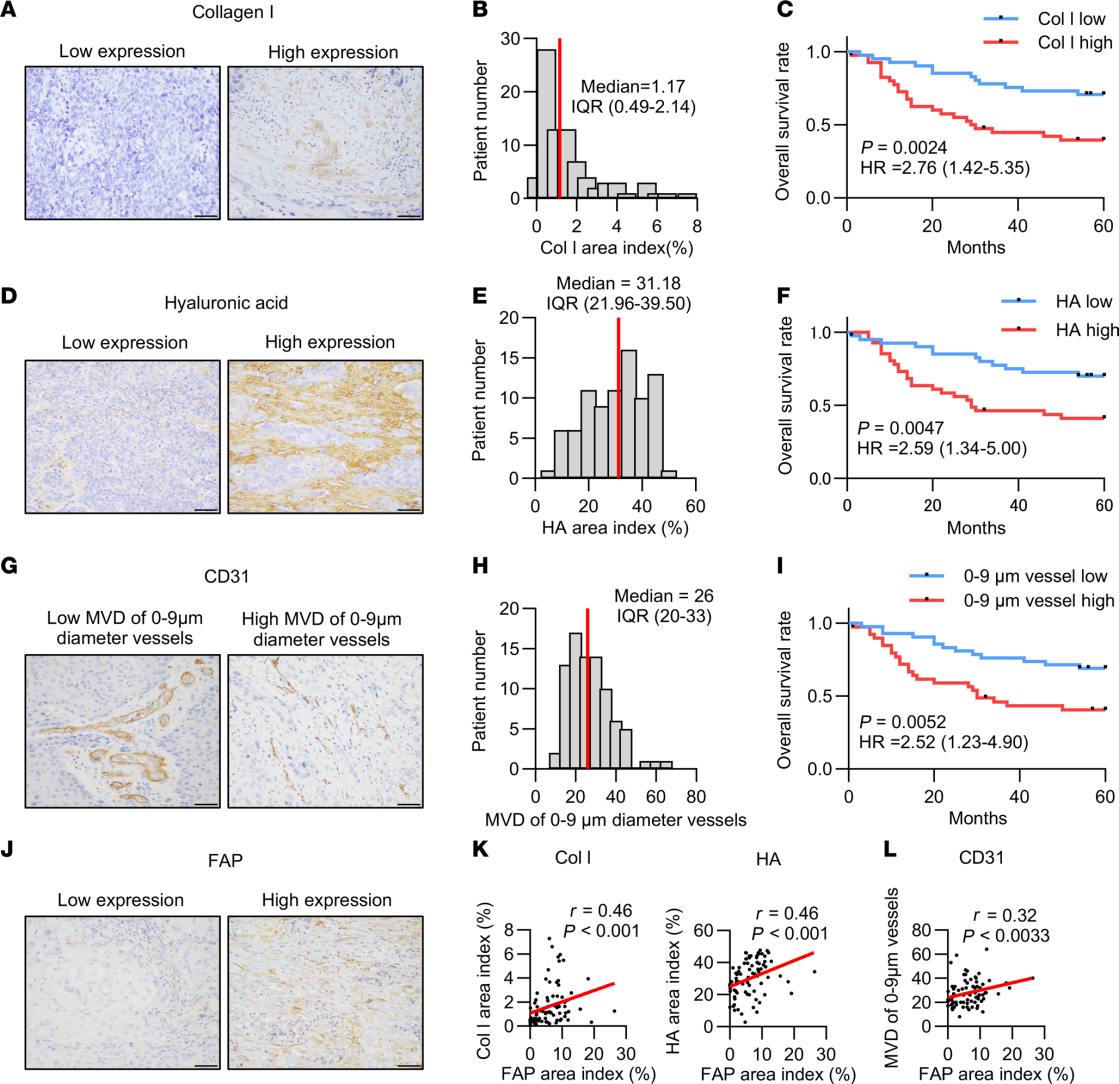
Correlation between CAF and ECM components, CD31 microvascular density, and clinical outcomes in esophageal cancer. (**A**) Representative esophageal cancer sample showing Col I staining (original magnification, ×400; scale bars: 50 μm). (**B**) Distribution of Col I area indices, with the median indicated (red bar). (**C**) Overall survival comparison between patients with high (*n* = 42) and low (*n* = 42) Col I levels (log-rank test). (**D**) Representative esophageal cancer sample stained for HA (original magnification, ×400; scale bars: 50 μm). (**E**) Histogram showing HA area indices, with the median marked (red bar). (**F**) Survival analysis for patients with high (*n* = 42) and low (*n* = 42) HA expression (log-rank test). (**G**) Representative esophageal cancer sample stained for CD31 (original magnification, ×400; scale bars: 50 μm). (**H**) Distribution of microvascular density (MVD) for vessels with diameters of 0–9 μm, with the median highlighted (red bar). (**I**) Survival comparison for patients with high (*n* = 41) and low (*n* = 43) MVD of 0 to 9 μm diameter vessels (log-rank test). (**J**) Representative esophageal cancer sample showing FAP staining (original magnification, ×400; scale bars: 50 μm). (**K**) Correlation between FAP levels and Col I/HA area indices in esophageal cancer samples (*n* = 84, Spearman’s correlation). (**L**) Correlation between FAP levels and MVD of 0 to 9 μm diameter vessels in esophageal cancer samples (*n* = 84, Spearman’s correlation).

Representative images of Col I expression are shown in Figure 2A at high magnification and Supplemental Figure 2A at low magnification. The positive site of Col I in IHC was calculated as an area index, as previously described (40). The 2 groups — high and low Col I expression — were defined based on the median value (Figure 2B). Survival analysis showed that patients with high Col I expression had a significantly poorer prognosis than those with low expression (Figure 2C). The clinicopathological characteristics according to Col I are summarized in Supplemental Table 1.

To evaluate the expression level of HA, representative IHC images are shown in Figure 2D at high magnification and Supplemental Figure 2B at low magnification. We evaluated the area index of HA expression and divided patients into 2 groups based on the median value (Figure 2E). Consistent with the Col I analysis, patients with high HA expression also had a significantly worse prognosis than those with low HA expression (Figure 2F).

Additionally, for vascular evaluation, we measured the MVD and the length of the longest diameter of the minor axis of each vessel by staining with CD31. The collapsed vessel was described as having a minor axis diameter of less than 10 μm, based on previous research (36), and representative IHC images are shown in Figure 2G. We measured the MVD of vessels with a 0 to 9 μm minor axis diameter and divided them into 2 groups based on the median value (Figure 2H). In survival analysis, patients with high MVD of 0–9 μm diameter had significantly worse prognosis compared with those with low MVD in the same size group (Figure 2I). The clinicopathological characteristics according to HA and MVD of 0 to 9 μm diameter vessels are summarized in Supplemental Table 2.

### ECM and MVD correlate with CAFs in esophageal cancer.

To investigate the relationship between ECM, vessel morphology, and CAF expression, we evaluated and quantified FAP expression, a specific marker for CAF, using resected tumors from the same cohort. Notably, FAP has previously been shown by our group to be associated with prognosis in esophageal cancer (13, 32). IHC images are shown in Figure 2J at high magnification and Supplemental Figure 2C at low magnification. When grouped according to the median value of the FAP expression area index (Supplemental Figure 2D), both the Col I and HA area indices were positively correlated with the FAP area index (Figure 2K). Moreover, the MVD of vessels with diameters of 0–9 μm was positively associated with FAP expression (Figure 2L). In contrast, the MVD of vessels with diameters of 10 μm or longer was negatively correlated with FAP (Supplemental Figure 2E). Patients with high MVD of 10 μm or larger in diameter had a better prognosis (Supplemental Figure 2F).

Building on these findings, we further assessed α-smooth muscle actin (αSMA), another marker of CAFs. IHC images and the median value of the αSMA expression area index are shown in Supplemental Figure 2, G and H. αSMA has previously been reported by our group to be associated with poor prognosis in esophageal cancer (14). In the present cohort, its expression showed a positive correlation with FAP (Supplemental Figure 2I). αSMA expression was significantly positively correlated with Col I, HA, and MVD of 0 to 9 μm diameter vessels, while αSMA expression had a significant negative correlation with MVD of 10 μm or larger diameter vessels (Supplemental Figure 2, J and K).

These results demonstrate a strong association between the presence of CAFs and elevated levels of ECM, Col I, and HA. Moreover, collapsed microvessels were significantly correlated with FAP expression in resected tumors and poor prognosis in patients with esophageal cancer.

### In vitro induction of Col I and HA by CAFs.

**Figure 3:**
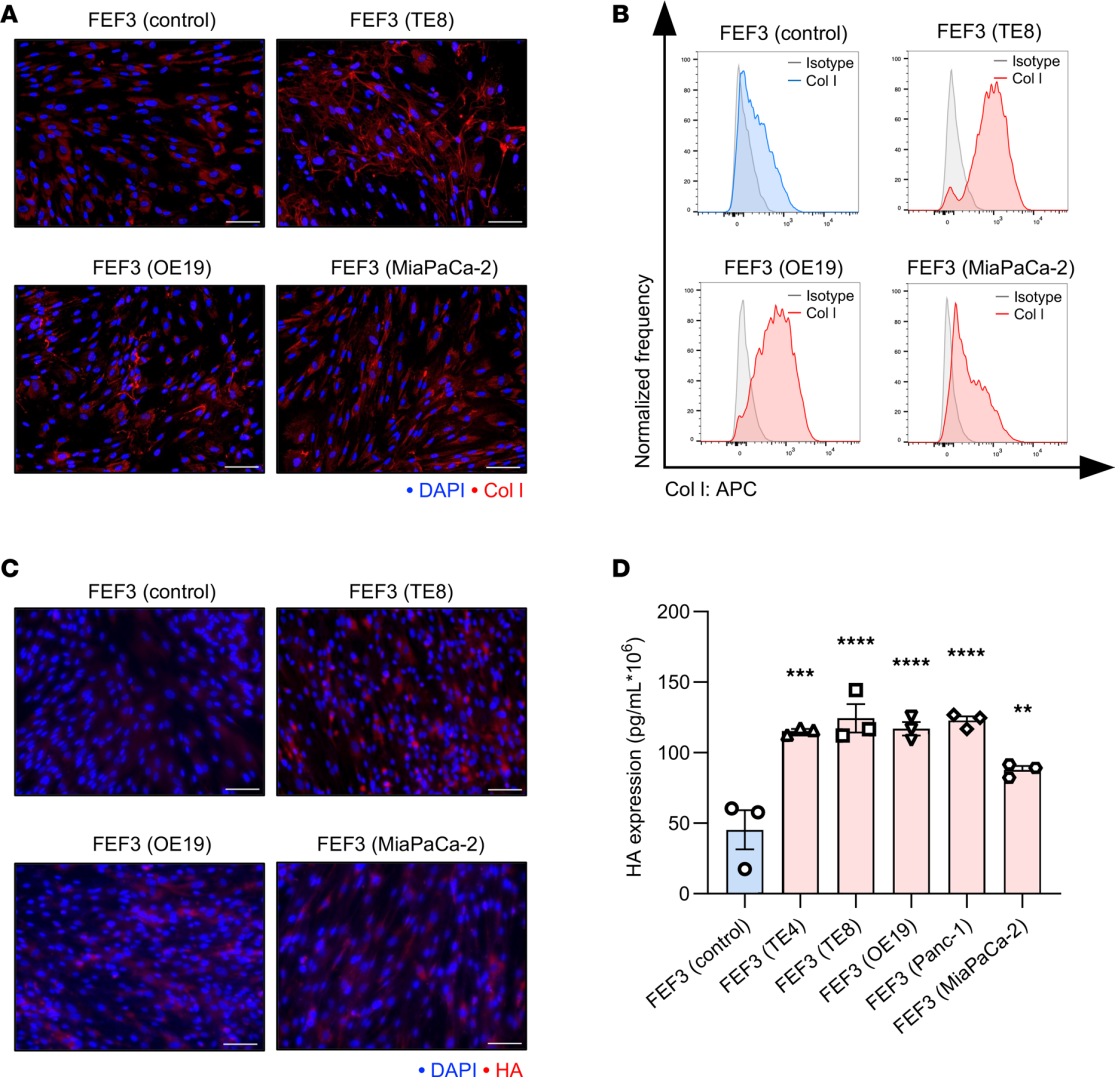
In vitro induction of Col I and HA by CAFs. (**A**) ICC images comparing Col I expression in FEF3 (control) and FEF3 (TE8, OE19, and MiaPaCa-2). Col I, red; DAPI, blue. Original magnification, ×200 (scale bars: 100 μm). (**B**) Histogram showing Col I expression levels in FEF3 (control) and FEF3 (TE8, OE19, and MiaPaCa-2). (**C**) ICC images comparing HA expression in FEF3 (control) and FEF3 (TE8, OE19, and MiaPaCa-2). HA, red; DAPI, blue. Original magnification, ×200 (scale bars: 100 μm). (**D**) ELISA results for HA in supernatants from FEF3 (control) and FEF3 (TE4, TE8, OE19, Panc-1, and MiaPaCa-2) (*n* = 3 per group; mean ± SEM; 1-way ANOVA with Tukey’s test). Values are normalized to cell count. Statistical significance: **P < 0.01; ***P < 0.001; ****P < 0.0001.

To evaluate the ability of CAFs to produce Col I or HA, CAFs stimulated by cancer cells (TE8, OE19, MiaPaCa-2, TE4, and Panc-1) were compared with normal fibroblasts (NFs) by immunocytochemistry (ICC). Fetal esophageal fibroblast 3 (FEF3) cells were used as NFs, as previously described (41). FEF3 (all cancer cells) exhibited heightened expression of Col I compared with FEF3 (control) in ICC. Figure 3A presents CAFs from TE8, OE19, and MiaPaCa-2, while Supplemental Figure 3A includes TE4 and Panc-1 to capture additional CAF diversity. In the quantitative analysis of Col I expression, flow cytometry (FCM) showed a significant increase in Col I expression in FEF3 (all cancer cells) compared with FEF3 (control) (Figure 3B and Supplemental Figure 3, B and C). FEF3 (all cancer cells) showed significantly higher FAP expression than FEF3 (control) (Supplemental Figure 3, D and E).

Similarly, HA deposition was greater in FEF3 (all cancer cells) compared with FEF3 (control) in ICC (Figure 3C). As with Col I, Supplemental Figure 3F provides additional information on CAF heterogeneity. ELISA further confirmed the significantly elevated HA expression in FEF3 (all cancer cells) compared with FEF3 (control) (Figure 3D). These results demonstrated CAFs’ involvement in the production of Col I and HA.

### Impact of CAFs on drug delivery in spheroid models.

**Figure 4:**
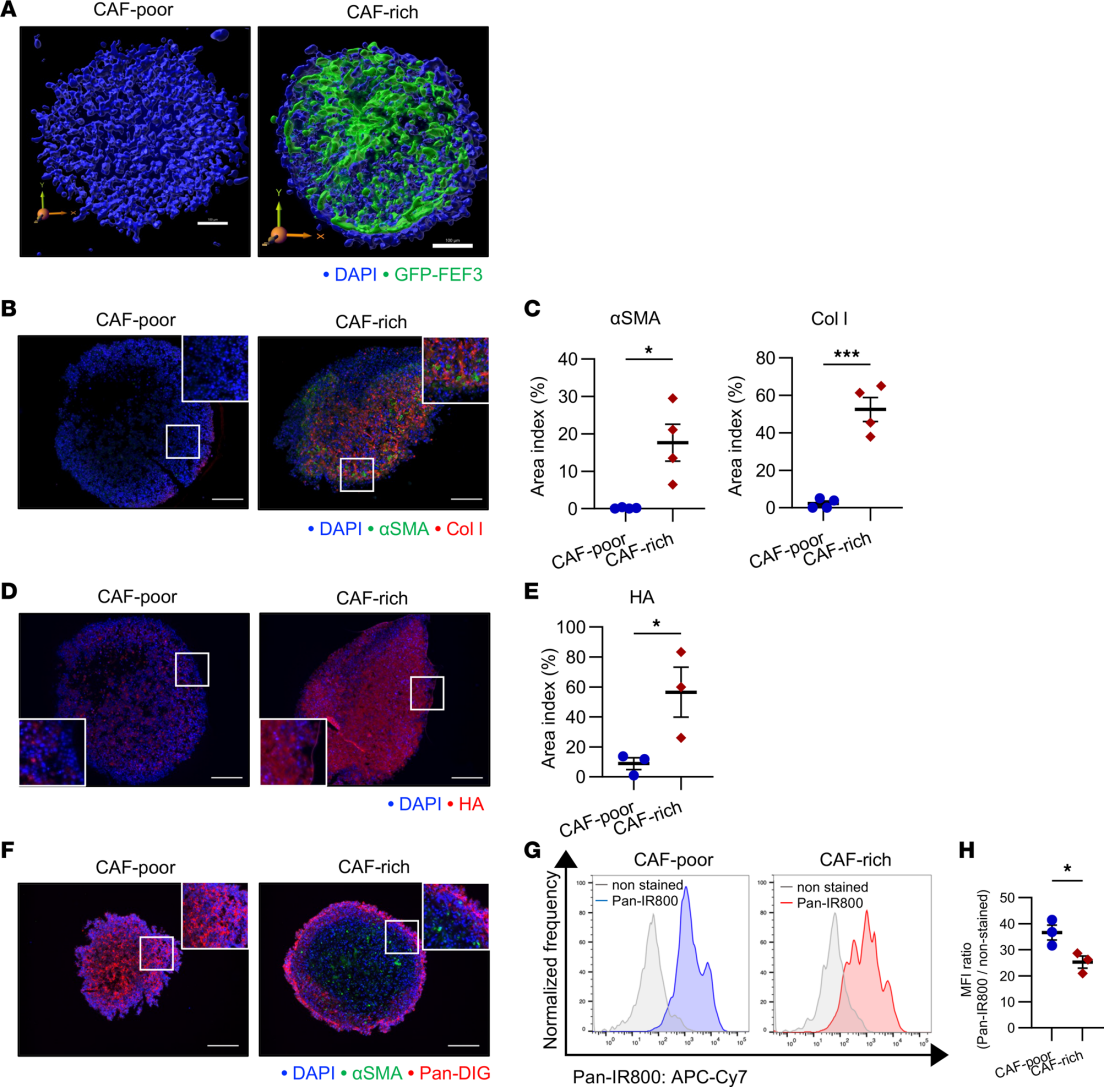
Impact of CAFs on drug delivery in spheroid models. CAF-poor spheroids (human TE8 cells only) and CAF-rich spheroids (human TE8 cells cocultured with human FEF3 cells at a 1:1 ratio) were used throughout this figure. (**A**) 3D visualization of spheroids: CAF-poor spheroid consisting only of TE8 cells, labeled with DAPI (blue); CAF-rich spheroid generated by coculturing of TE8 and GFP-FEF3 cells, labeled with GFP-FEF3 (green) and DAPI (blue) (scale bars: 100 μm). (**B**) Fluorescent IHC comparing αSMA (green), Col I (red), and DAPI (blue) in CAF-poor and CAF-rich spheroids at ×100 original magnification (scale bars: 200 μm). (**C**) Analysis of αSMA- and Col I–positive area indices between CAF-poor and CAF-rich spheroids (*n* = 4; mean ± SEM; unpaired *t* test). (**D**) Fluorescent IHC showing HA (red) and DAPI (blue) in CAF-poor and CAF-rich spheroids at ×200 original magnification (scale bars: 100 μm). (**E**) Comparison of HA-positive area indices between CAF-poor and CAF-rich spheroids (*n* = 3; mean ± SEM; unpaired *t* test). (**F**) Fluorescent IHC illustrating drug penetration using Pan-DIG in CAF-poor and CAF-rich spheroids. αSMA, green; DIG, red; DAPI, blue. Original magnification, ×100 (scale bars: 200 μm). (**G**) Histogram depicting Pan-IR800 accumulation in TE8 cells under CAF-poor and CAF-rich conditions. (**H**) Quantitative assessment of Pan-IR800 uptake in TE8 cells between CAF-poor and CAF-rich spheroids (*n* = 3 independent experiments; mean ± SEM; unpaired *t* test). Statistical significance: *P < 0.05; ***P < 0.001.

To assess the effects of CAFs on drug delivery, we developed 3D in vitro models. A spheroid composed solely of TE8 cells was generated to represent the CAF-poor model, while a cocultured spheroid containing TE8 cells and FEF3 fibroblasts at a 1:1 ratio was generated to represent the CAF-rich model. Immunofluorescence confirmed that the CAF-poor model was composed solely of TE8 cells, whereas the CAF-rich model exhibited a uniform distribution of TE8 and FEF3 throughout the spheroid in a 3D coculture (Figure 4A). Supplemental Video 1 (CAF-poor model) and Supplemental Video 2 (CAF-rich model) are 3D videos, further illustrating the spatial distribution of TE8 and FEF3 within the spheroid. When stained with cytokeratin, which indicates cancer cells, the staining extended to the central area (Supplemental Figure 4A). Together with Figure 4A and Supplemental Figure 4A, the CAF-rich spheroids appeared to be a relatively uniform spherical model. To evaluate the ECM and CAF in spheroids, the Col I, HA, and αSMA expression levels were compared between CAF-rich and CAF-poor groups using fluorescent IHC. CAF-rich models had significantly higher Col I and αSMA levels (Figure 4, B and C). Similarly, HA levels in CAF-rich models were significantly higher than those in CAF-poor models (Figure 4, D and E). To assess drug accumulation in the spheroids, the binding rate of digoxigenin-labeled panitumumab (Pan-DIG) to the EGFR of TE8 cells was investigated. Almost 100% of EGFR was expressed on the surface antigen of TE8 (Supplemental Figure 4, B and C). We injected Pan-DIG into both CAF-poor and CAF-rich models, and observed the binding rate of Pan-DIG 6 hours later using fluorescent IHC. In the CAF-rich model, immunofluorescence indicated that Pan-DIG was poorly bound to cancer cells in the spheroid core compared with the CAF-poor model (Figure 4F).

To quantitatively evaluate drug accumulation in the spheroids, the binding of panitumumab to TE8 cancer cells in the CAF-poor and CAF-rich models was evaluated using FCM. FEF3 in the CAF-rich model was removed by staining with UltraGreen in FCM. The gating strategy is shown in Supplemental Figure 4, D and E. FCM revealed that TE8 cells in the CAF-rich model showed significantly lower binding to panitumumab than those in the CAF-poor model (Figure 4, G and H).

These findings suggest that binding of panitumumab to EGFR-expressing cancer cells in the spheroid model is inhibited by coculture with CAFs.

### Efficacy of FAP-targeted NIR-PIT in CAF-rich spheroid models.

**Figure 5:**
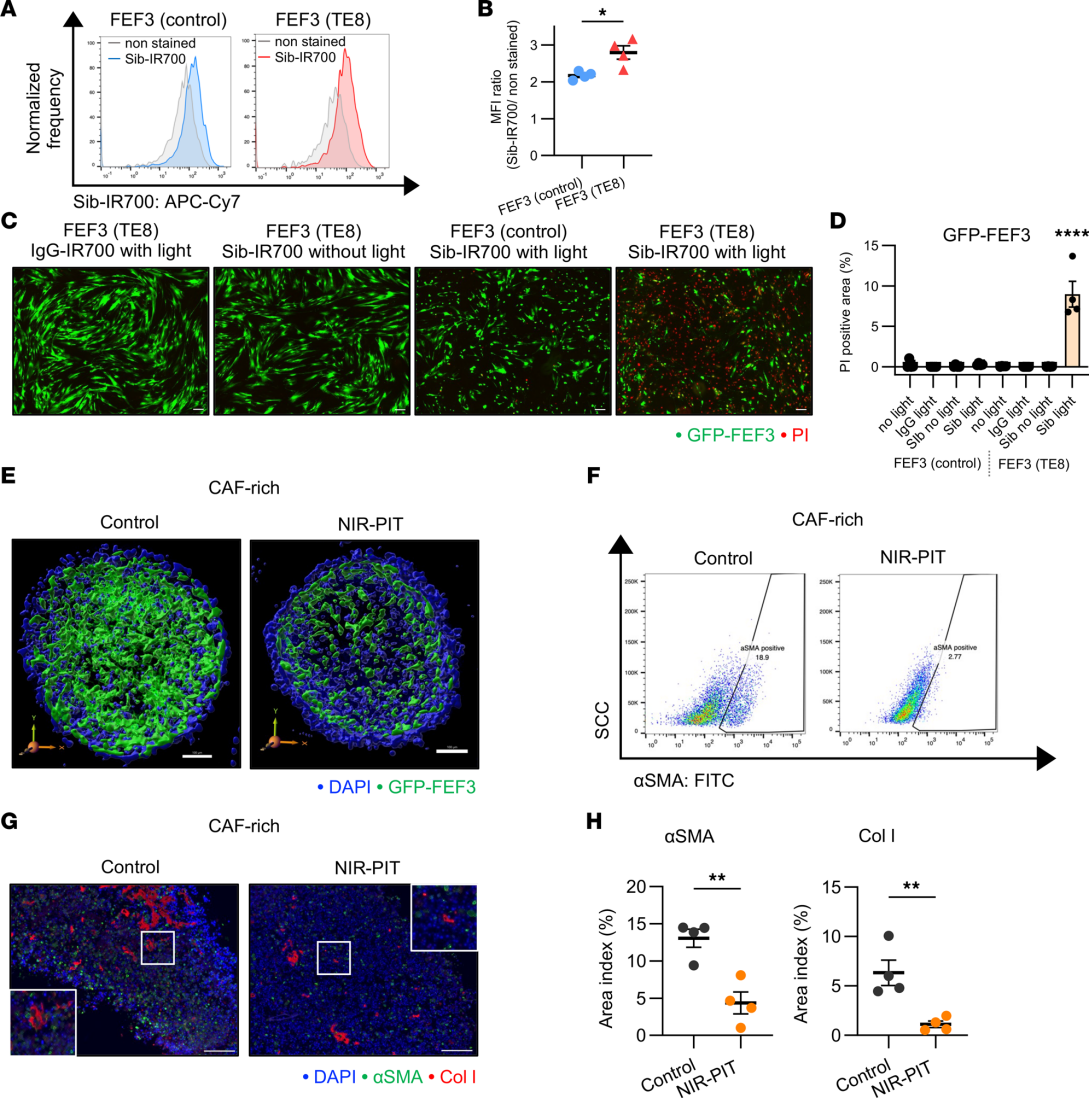
Efficacy of FAP-targeted NIR-PIT in CAF-rich spheroid models. CAF-rich spheroids (human TE8 cells cocultured with human FEF3 cells at a 1:1 ratio) were used throughout this figure. (**A**) Histogram illustrating Sib-IR700 binding in FEF3 (control) and FEF3 (TE8). (**B**) Quantitative analysis of Sib-IR700 binding between FEF3 (control) and FEF3 (TE8) (*n* = 4; mean ± SEM; unpaired *t* test). (**C**) Fluorescent images of PI staining in FEF3 (control) and FEF3 (TE8) treated with IgG-IR700 or Sib-IR700, with or without NIR. GFP-FEF3, green; PI, red. Original magnification, ×40 (scale bars: 200 μm). (**D**) Comparative analysis of PI-positive area indices across groups: untreated, IgG-IR700 or Sib-IR700, with or without NIR (*n* = 4 per group; mean ± SEM; 1-way ANOVA with Tukey’s test). (**E**) 3D visualization of untreated CAF-rich control spheroids and NIR-PIT–treated CAF-rich spheroids. GFP-FEF3, green; DAPI, blue. (**F**) Flow cytometric analysis showing αSMA-positive cells reduced from 18.9% (control) to 2.77% (NIR-PIT–treated) in CAF-rich spheroids 2 days after treatment. (**G**) Fluorescent IHC images of αSMA and Col I in control and NIR-PIT–treated CAF-rich spheroids 2 days after treatment. αSMA, green; Col I, red; DAPI, blue. Original magnification, ×100 (scale bars: 200 μm). (**H**) Quantitative comparison of αSMA and Col I area indices (percent) between control and NIR-PIT–treated CAF-rich spheroids (*n* = 4; mean ± SEM; unpaired *t* test). Statistical significance: *P < 0.05; **P < 0.01; ****P < 0.0001.

In this study, we successfully conjugated the anti–human FAP antibody sibrotuzumab to the photoabsorber IRDye700DX *N*-hydroxysuccinimide ester (IR700) (Supplemental Figure 5A). To confirm the binding of IR700-conjugated sibrotuzumab (Sib-IR700) to FAP in CAFs, FCM analysis was performed in vitro. Compared with FEF3 (control), Sib-IR700 bound significantly more to FEF3 (TE8) (Figure 5, A and B), consistent with our previous study (32). To assess the cytotoxicity of FAP-targeted NIR-PIT, immunofluorescence microscopy with propidium iodide (PI) staining was performed using GFP-labeled FEF3 cells. The efficacy of NIR-PIT was significantly higher in FEF3 (TE8). Neither NIR light nor Sib-IR700 alone significantly affected cell viability. Moreover, NIR-PIT cytotoxicity was not detected in FEF3 (control) (Figure 5, C and D). Consistently, ICC confirmed binding specificity: Sib-IR700 bound selectively to FEF3 (TE8) and induced PI uptake after NIR-PIT, whereas IgG-IR700 showed no binding in either cell type (Supplemental Figure 5B). Similar effects were observed in WI-38 cells, another human fibroblast line (Supplemental Figure 5, C and D).

To investigate the clinical effects of FAP-targeted NIR-PIT in CAF-rich tumors, we monitored changes in the internal structure of a CAF-rich model after treatment using 3D imaging. Fluorescence imaging revealed a notable reduction in fibroblast numbers 1 hour after FAP-targeted NIR-PIT (Figure 5E). Supplemental Video 3 (Control) and Supplemental Video 4 (NIR-PIT) are in 3D, further illustrating the fibroblast reduction following NIR-PIT. We quantitatively evaluated αSMA and Col I expression in these spheroid models 2 days after FAP-targeted NIR-PIT. In CAF-rich spheroid models, αSMA expression in whole live cells was significantly reduced 2 days after NIR-PIT (Figure 5F and Supplemental Figure 6A). The gating strategy is shown in Supplemental Figure 6B.

Furthermore, we evaluated αSMA and Col I expression in these spheroid models 2 days after FAP-targeted NIR-PIT using fluorescent IHC (Figure 5G) and H&E/IHC for additional histopathological validation (Supplemental Figure 6C). This result revealed a significant decrease in αSMA and Col I secretion in the NIR-PIT group compared with the control group (Figure 5H), suggesting that FAP-targeted NIR-PIT reduces both the CAF and ECM populations.

### Impact of FAP-targeted NIR-PIT on drug delivery in CAF-rich spheroid models.

To assess the impact of FAP-targeted NIR-PIT on drug accumulation in CAF-rich spheroid models, we focused on panitumumab, a monoclonal antibody, and Abraxane, an albumin-bound form of paclitaxel. Previous studies have shown that SUPR enhances the EPR effect, particularly by improving the delivery of high–molecular weight drugs (27, 28, 42, 43). Therefore, we selected Abraxane, as paclitaxel is a clinically approved agent for treating esophageal cancer.

**Figure 6:**
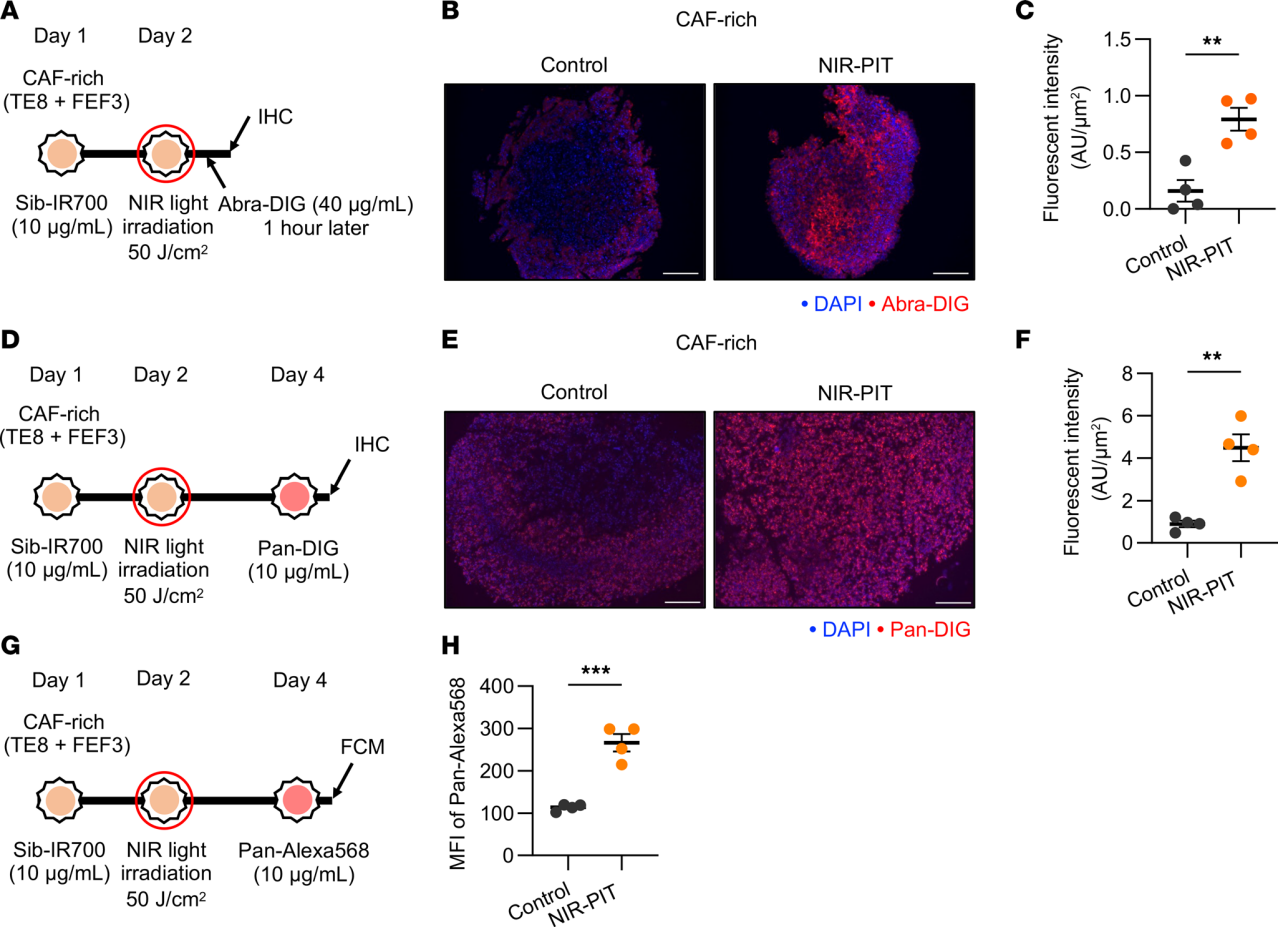
Impact of FAP-targeted NIR-PIT on drug delivery in CAF-rich spheroid models. CAF-rich spheroids (human TE8 cells cocultured with human FEF3 cells at a 1:1 ratio) were used throughout this figure. (**A**) Diagram of treatment schedule and NIR light irradiation for IHC in FAP-targeted NIR-PIT. (**B**) Fluorescent IHC images showing Abra-DIG distribution in control and NIR-PIT–treated CAF-rich spheroids 1 hour after treatment. DIG, red; DAPI, blue. Original magnification, ×100 (scale bars: 200 μm). (**C**) Quantitative analysis of Abra-DIG fluorescence intensity per area (μm^2^) in control and NIR-PIT–treated CAF-rich spheroids (*n* = 4; mean ± SEM; unpaired *t* test). (**D**) Protocol diagram for treatment schedule and NIR light irradiation in IHC. (**E**) Fluorescent IHC images depicting Pan-DIG distribution in control and NIR-PIT–treated CAF-rich spheroids 2 days after treatment. DIG, red; DAPI, blue. Original magnification, ×100 (scale bars: 200 μm). (**F**) Quantitative comparison of Pan-DIG fluorescence intensity per area (μm^2^) in control and NIR-PIT–treated CAF-rich spheroids (*n* = 4; mean ± SEM; unpaired *t* test). (**G**) Diagram of treatment schedule and NIR light irradiation protocol for flow cytometric analysis. (**H**) Flow cytometric analysis quantifying Pan-Alexa568 binding in control and NIR-PIT–treated CAF-rich spheroids (*n* = 4; mean ± SEM; unpaired *t* test). Statistical significance: **P < 0.01; ***P < 0.001.

Initially, drug accumulation was measured 1 hour after FAP-targeted NIR-PIT using Pan-DIG and digoxigenin-labeled Abraxane (Abra-DIG), under the same experimental conditions as in the experiment shown in Figure 5E. Based on previous studies, the protocol is shown in Supplemental Figure 6D and Figure 6A (27). Multiplex IHC showed that Pan-DIG and Abra-DIG bound to cancer cells in the core region of the NIR-PIT group (Figure 6B and Supplemental Figure 6E). Quantitative analysis using the area index revealed that the total fluorescence intensity of Pan-DIG and Abra-DIG in the NIR-PIT group was significantly higher than that in the control group (Figure 6C and Supplemental Figure 6F).

Next, we evaluated drug accumulation 2 days after FAP-targeted NIR-PIT, which demonstrated a reduction in CAFs and ECM, as shown in Figure 5, G and H, and Supplemental Figure 6C. This protocol is shown in Figure 6D. There was also a significant increase in Pan-DIG in the NIR-PIT group (Figure 6, E and F). Additionally, to quantitatively evaluate the binding rate of Pan-DIG, whole live cells of CAF-rich spheroids were analyzed using FCM (Figure 6G). The results showed that panitumumab binding to TE8 by FAP-targeted NIR-PIT was significantly enhanced (Figure 6H).

These results demonstrated that FAP-targeted NIR-PIT improved drug accumulation in CAF-rich spheroids.

### In vivo enhanced drug delivery of panitumumab and Abraxane by FAP-targeted NIR-PIT.

**Figure 7:**
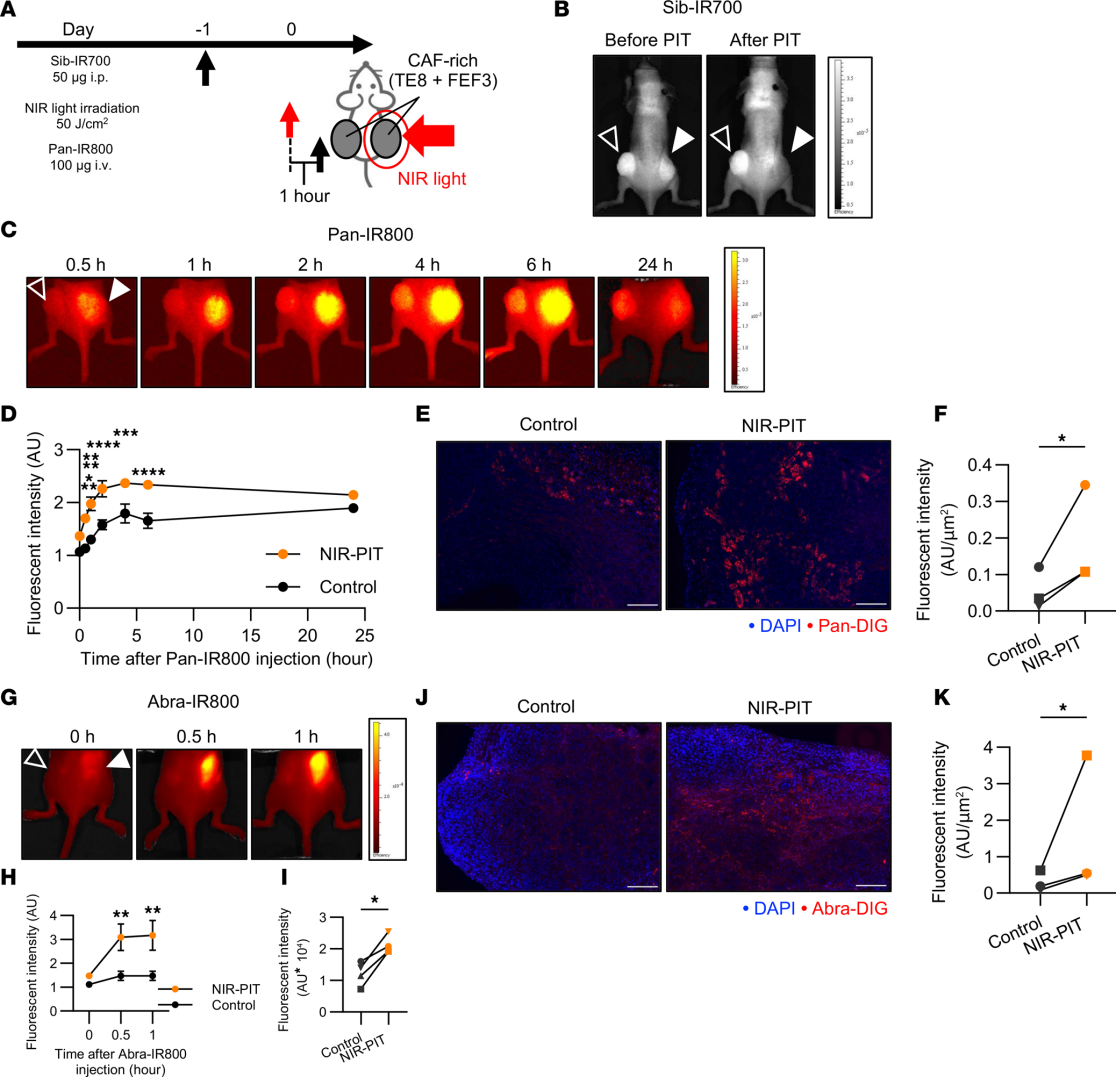
In vivo enhanced drug delivery of panitumumab and Abraxane by FAP-targeted NIR-PIT. CAF-rich tumors (human TE8 cells cocultured with human FEF3 cells at a 1:3 ratio) were used throughout this figure. (**A**) Schematic of treatment protocol and NIR light exposure. (**B**) Sib-IR700 imaging before and after FAP-targeted NIR-PIT in a bilateral CAF-rich tumor model with tumors reaching 500 mm^3^. Filled arrowheads indicate NIR-irradiated tumors (right), and open arrowheads indicate non-irradiated tumors (left). (**C**) Pan-IR800 imaging in a bilateral CAF-rich tumor model following FAP-targeted NIR-PIT. Filled arrowheads show NIR-irradiated tumors (right), and open arrowheads show non-irradiated tumors (left). (**D**) Fluorescence intensity ratio analysis (control/background vs. NIR-PIT–treated/background) over time after Pan-IR800 administration (*n* = 4; mean ± SEM; repeated-measures 2-way ANOVA with Tukey’s test). (**E**) Fluorescent IHC image showing Pan-DIG accumulation in control versus NIR-PIT–treated tumors 1 hour after treatment. DIG, red; DAPI, blue. Original magnification, ×100 (scale bars: 200 μm). (**F**) Quantitative comparison of Pan-DIG fluorescence intensity per area (μm^2^) between control and NIR-PIT–treated tumors (*n* = 3; ratio paired *t* test). (**G**) Abra-IR800 imaging in bilateral CAF-rich tumor models after FAP-targeted NIR-PIT. Filled arrowheads indicate NIR-irradiated tumors (right), and open arrowheads indicate non-irradiated tumors (left). (**H**) Fluorescent intensity ratio analysis (control/background vs. PIT-treated/background) after Abra-IR800 application (*n* = 4; mean ± SEM; repeated-measures 2-way ANOVA with Tukey’s test). (**I**) Comparative analysis of mean fluorescence intensity in control and NIR-PIT–treated tumors for each mouse (*n* = 4; ratio paired *t* test). (**J**) Fluorescent IHC image showing Abra-DIG accumulation in control versus NIR-PIT–treated tumors 1 hour after treatment. DIG, red; DAPI, blue. Original magnification, ×100 (scale bars: 200 μm). (**K**) Quantitative comparison of Abra-DIG fluorescence intensity per area (μm^2^) between control and NIR-PIT–treated tumors (*n* = 3; ratio paired *t* test). Statistical significance: *P < 0.05; **P < 0.01; ***P < 0.001; ****P < 0.0001.

To assess drug delivery in CAF-rich tumors in vivo, we used a bilateral subcutaneous tumor model in the flank. The treatment schedule is outlined in Figure 7A. Briefly, only the tumors on the right flank were irradiated with NIR light, while the left-sided tumor was completely covered with aluminum foil during light exposure to block any light absorption 1 day after Sib-IR700 injection. We intravenously administered IRDye800CW *N*-hydroxysuccinimide ester–conjugated (IR800-conjugated) panitumumab (Pan-IR800) 1 hour after NIR-PIT.

We injected Sib-IR700 into tumors that reached 500 mm^3^. Sib-IR700–injected mouse tumors exhibited 700 nm fluorescent signals at both tumor sites before therapeutic NIR light exposure. Immediately after irradiation of the right tumor with NIR light, the signal disappeared, suggesting photobleaching of Sib-IR700 (Figure 7B). Eight-hundred-nanometer near-infrared fluorescence (NIRF) imaging showed that Pan-IR800 was significantly increased in NIR-PIT–treated tumors, specifically in the early stages, compared with untreated tumors (Figure 7, C and D). Furthermore, for smaller tumors reaching 100 mm^3^, FAP-targeted NIR-PIT significantly enhanced drug delivery (Supplemental Figure 7, A–C). For ex vivo analysis, we intravenously injected Pan-DIG after FAP-targeted NIR-PIT and sacrificed the mice to evaluate the Pan-DIG distribution at the tumor site (Supplemental Figure 7D). This revealed that FAP-targeted NIR-PIT significantly increased Pan-DIG binding to the tumor site (Figure 7, E and F).

Changes in Abraxane accumulation were assessed using FAP-targeted NIR-PIT. Following the protocol (Supplemental Figure 8A), FAP-targeted NIR-PIT was performed only on the right-sided tumors in a bilateral model. The accumulation of IR800-conjugated Abraxane (Abra-IR800) in NIR-PIT–treated tumors was enhanced in comparison with control tumors (Figure 7, G and H, and Supplemental Figure 8B).

To eliminate the effect of skin or tumor depth on fluorescence intensity measurements using NIRF imaging, tumors were harvested, and the fluorescence intensity of Abra-IR800 was directly measured by NIRF imaging. The intensity of Abra-IR800 was significantly higher in the NIR-PIT group than in the control group (Figure 7I), as illustrated by representative imaging in Supplemental Figure 8C. To confirm the distribution of Abraxane in the tumor, multiplex IHC was performed ex vivo (Supplemental Figure 8D). The fluorescence intensity of Abra-DIG in the FAP-targeted NIR-PIT group was significantly higher at the tumor site (Figure 7, J and K).

Overall, FAP-targeted NIR-PIT not only enhanced the drug delivery of panitumumab and Abraxane in CAF-rich tumors at the macroscopic level but also demonstrated significant enhancement at the microscopic level.

### In vivo synergistic antitumor effects of FAP-targeted NIR-PIT combined with Abraxane.

**Figure 8:**
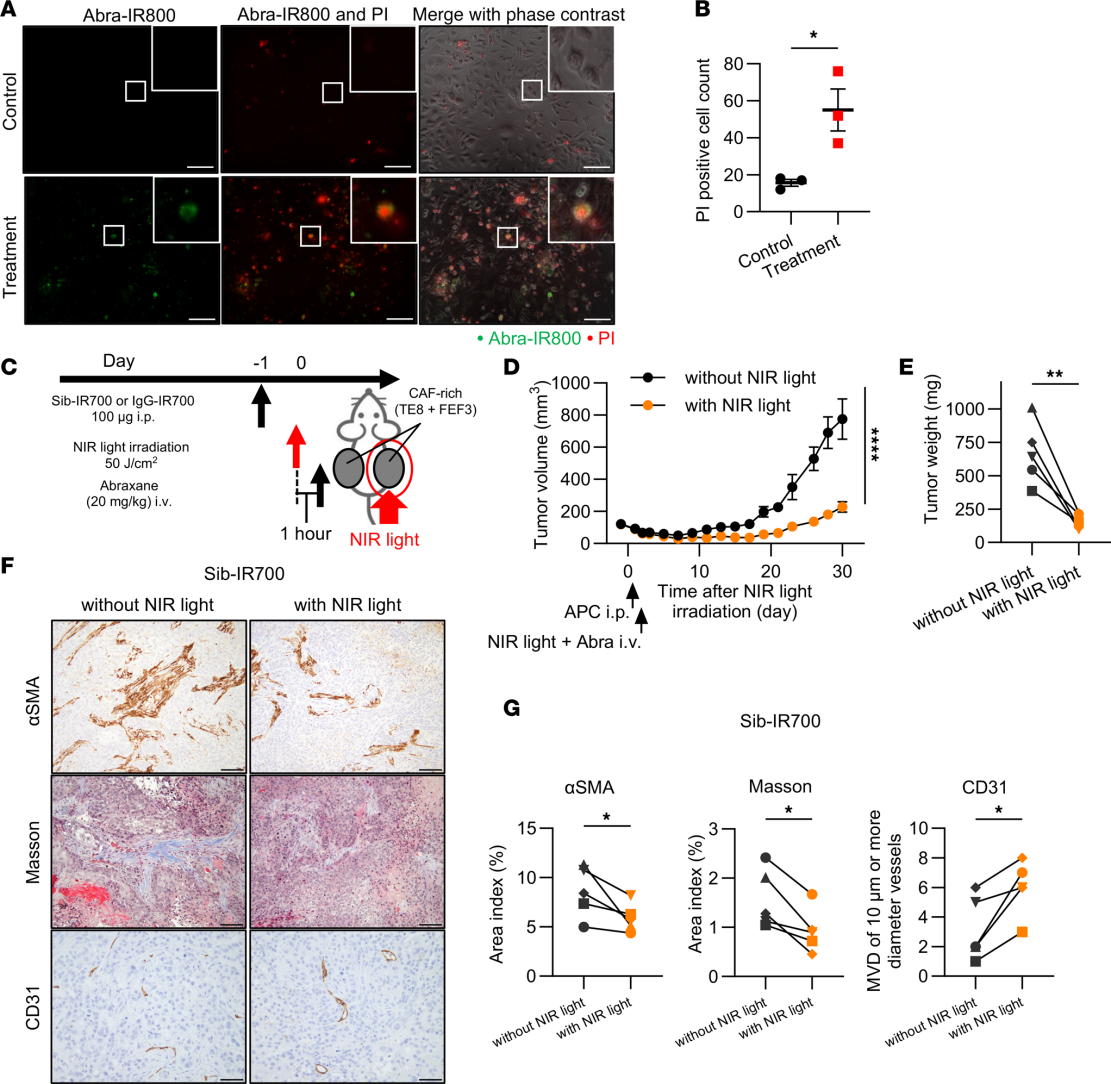
In vivo synergistic antitumor effects of FAP-targeted NIR-PIT combined with Abraxane. CAF-rich tumors (human TE8 cells cocultured with human FEF3 cells at a 1:3 ratio) were used throughout this figure. (**A**) Fluorescent images of PI staining in TE8 cells, untreated (control) or treated with Abra-IR800. Abra-IR800, green; PI, red. Original magnification, ×200 (scale bars: 100 μm). (**B**) Quantitative analysis of PI-positive cell counts in untreated (control) and Abra-IR800–treated cells (*n* = 3; mean ± SEM; unpaired *t* test). (**C**) Diagram of treatment protocol and NIR light application. (**D**) Tumor growth curve in mice injected with Sib-IR700, with or without NIR light exposure (*n* = 5; mean ± SEM; 1-way ANOVA with Tukey’s test). (**E**) Comparison of tumor weights between right-side tumors exposed to NIR light and left-side tumors without NIR light in Sib-IR700–injected mice (*n* = 5; ratio paired *t* test). (**F**) IHC images showing αSMA, Masson’s trichrome staining, and CD31 in tumors treated with Sib-IR700, with or without NIR light. αSMA and Masson’s trichrome staining, original magnification ×200 (scale bars: 100 μm); CD31, original magnification ×400 (scale bars: 50 μm). (**G**) Quantitative comparison of αSMA and Masson’s trichrome staining areas, as well as MVD of vessels at least 10 μm in diameter, in tumors treated with Sib-IR700 without versus with NIR light (*n* = 5; ratio paired *t* test). Statistical significance: *P < 0.05; **P < 0.01; ****P < 0.0001.

We first confirmed that Abraxane induced cytotoxicity in TE8 cells in vitro (Figure 8, A and B). Abraxane significantly suppressed tumor progression in the TE8 xenograft models (CAF-poor models) (Supplemental Figure 9A). However, in xenografts co-inoculated with TE8 and FEF3, the CAF-rich models, Abraxane did not have a significant effect (Supplemental Figure 9B).

To improve the efficacy of Abraxane in CAF-rich tumors, we performed a combined treatment with Abraxane and FAP-targeted NIR-PIT on bilateral tumors cocultured with TE8 and FEF3 cells. The treatment regimen is shown in Figure 8C. Only the right-sided tumors were exposed to NIR light, whereas the left-sided tumors were completely covered and not exposed to NIR light. One hour after light irradiation, Abraxane was administered intravenously.

In the Sib-IR700 group, FAP-targeted NIR-PIT combined with Abraxane significantly inhibited CAF-rich tumor progression (Figure 8D). No significant differences were detected in the IR700-conjugated isotype control (IgG-IR700) group (Supplemental Figure 10A). The mice were euthanized when the tumor volume reached 1,000 mm^3^. The harvested tumor weights in the NIR-PIT and Abraxane groups were significantly lower than those in tumors without NIR light (Figure 8E), as visually demonstrated in Supplemental Figure 10B. In the IgG-IR700 group, there was no significant difference (Supplemental Figure 10, C and D). In addition, there was no significant difference in body weight between the Sib-IR700 and the IgG-IR700 groups, suggesting that there were no serious adverse effects (Supplemental Figure 10E). In IHC analysis of harvested tumors in the Sib-IR700 group, there was a significant reduction of αSMA and collagen in tumors with NIR light compared with tumors without NIR light (Figure 8, F and G). Additionally, MVD of vessels of 10 μm or more diameter significantly increased in tumors with NIR light (Figure 8, F and G).

These results indicated that FAP-targeted NIR-PIT combined with Abraxane induced antitumor effects compared with Abraxane treatment alone. Furthermore, in IHC, FAP-targeted NIR-PIT contributed to reducing αSMA and collagen and changing vessel morphology.

## Discussion

This study demonstrated that the presence of cancer-associated fibroblasts (CAFs) in CAF-rich models significantly impairs drug delivery. However, FAP-targeted NIR-PIT improved drug delivery in both in vitro and in vivo CAF-rich models by specifically targeting and reducing FAP-positive CAFs. Additionally, enhanced drug accumulation resulting from this approach led to a more pronounced antitumor effect, especially when combined with chemotherapy.

Our results showed that abundant CAFs reduced drug delivery in both 3D in vitro and in vivo models. In CAF-rich environments, structural changes such as vascular compression, collapse, and reduced blood flow were observed, suggesting that the increased cellular density and ECM production in CAF-rich tumors physically compress the vasculature, thus impeding drug delivery. Specifically, elevated Col I and HA levels identified in this study likely contribute to tumor stiffening by creating functional barriers that reduce vascular permeability (44, 45). These observations align with clinical findings, where elevated Col I and HA levels have been linked to poor prognosis, further reinforcing our conclusions.

FAP-targeted NIR-PIT effectively diminished FAP-positive CAFs in both 3D in vitro and in vivo models. This therapeutic approach utilizes the conjugation of sibrotuzumab to the IR700 dye. Upon NIR exposure, structural changes in IR700 induce membrane rupture and subsequent necrosis of targeted cells. A recent study has further clarified that this unique mode of cell death, termed “photochemosis,” is mediated by the rapid cortical actin network disruption following IR700 aggregation, leading to catastrophic membrane rupture distinct from apoptosis (46). This instantaneous mechanism explains the rapid effect of FAP-targeted NIR-PIT.

Importantly, this method demonstrated high specificity for CAFs, with minimal impact on NFs. In vivo, FAP-targeted NIR-PIT showed efficacy against CAF-rich tumors, leading to a tumor growth curve comparable to that observed in CAF-poor tumors, indicating its potential as a promising CAF-targeted therapeutic strategy (15, 30–32). Although some CAFs remained after the treatment shown in Figures 5 and 8 — likely owing to limited light exposure in a single treatment in this study, CAF heterogeneity, or stromal repopulation — further investigation is warranted.

FAP-targeted NIR-PIT significantly improved drug delivery, primarily as a result of the SUPR effect. This mechanism, previously reported for EGFR-targeted NIR-PIT (27), involves rapid CAF necrosis that reduces cell density and relieves external pressure on blood vessels, thereby enhancing intratumoral blood flow. While this approach is effective, drug penetration into the tumor can still be hindered by the presence of CAF-rich environments. Previous studies have shown that abundant CAFs around blood vessels obstruct nanoparticle delivery to the tumor core (47, 48). Nevertheless, FAP-targeted NIR-PIT enhanced SUPR effects in CAF-rich tumors, as evidenced in this study. One possible explanation for the strong SUPR effect of FAP-targeted NIR-PIT in CAF-rich environments is the localization of CAFs within or around the vasculature. Previous research has shown that certain CAF subtypes, such as vascular CAFs or FAP-positive fibroblasts, are located in perivascular regions or even originate from pericytes (49–51). The immediate diminution of these perivascular CAFs by photochemosis is likely to dismantle stromal barriers, induce vascular dilation, and reduce interstitial pressure, thereby facilitating enhanced drug penetration. Therefore, it is plausible that the administered Sib-IR700 directly targeted and effectively damaged CAFs, which contributed to the therapeutic effects observed in the present study.

Another contributing factor may be the suppression of the functional role of collagen by FAP-targeted NIR-PIT, which may have further enhanced drug delivery. Collagen attaches to DDR1 receptors on tumor cells. This activation strengthens collagen structures and creates barriers that make it harder for drugs and immune cells to penetrate (52). The observed reduction in collagen levels following FAP-targeted NIR-PIT may therefore have contributed to improved drug delivery, potentially through reduced DDR1 activation. However, this mechanism was not directly examined and warrants further investigation.

Moreover, the combination of FAP-targeted NIR-PIT with Abraxane maintained vascular integrity and normalized vessel diameters, potentially because of reduced intratumoral pressure and CAF reduction (21, 53). These findings suggest that ECM remodeling and vascular normalization synergistically enhance drug delivery.

The combination of FAP-targeted NIR-PIT with Abraxane resulted in enhanced therapeutic effects, mainly due to improved drug delivery through the SUPR effect. This approach allows drugs to penetrate the tumor. In addition to the SUPR effect, FAP-targeted NIR-PIT has been shown to improve tumor-infiltrating lymphocytes and activate antitumor immunity (33). Given this, combining FAP-targeted NIR-PIT with immune checkpoint inhibitors (ICIs) may enhance ICI antibody delivery, leading to a more potent therapeutic response. Although our study used a single NIR light exposure to isolate the drug delivery improvement effects, multiple exposures and antibody administrations, as reported in prior studies, may further improve outcomes (54, 55). Optimizing dosing intervals and administration frequencies could intensify these effects, providing the opportunity for personalized treatment strategies based on tumor and patient characteristics. Furthermore, the use of sibrotuzumab, a humanized antibody that has been validated in clinical trials, supports the potential for FAP-targeted NIR-PIT to be implemented in clinical settings (24, 32).

This study had some limitations. Firstly, the subcutaneous tumor models used may not completely replicate the stromal composition and microenvironment characteristics found in clinical tumors. Secondly, while the study demonstrated enhanced drug delivery, direct measurements of intratumoral or interstitial pressure changes were not conducted; we relied on indirect evidence in this regard. Thirdly, although reductions in ECM and improved drug delivery were observed, the precise mechanisms behind ECM remodeling remain unclear and merit further investigation. Fourthly, the long-term intratumoral distribution of Abraxane after NIR-PIT could not be directly evaluated. Lastly, only a few drugs were evaluated in this study, and further exploration of other therapeutic agents is needed to fully assess the potential of FAP-targeted NIR-PIT.

In conclusion, this study highlights CAF-rich tumor environments as a significant barrier to drug delivery and demonstrates the potential of FAP-targeted NIR-PIT as an effective strategy to overcome this challenge. By selectively reducing CAFs, FAP-targeted NIR-PIT induces the SUPR effect, leading to improved tumor blood flow, ECM reduction, and enhanced drug delivery. The combination of this therapy with chemotherapy results in robust antitumor effects. Furthermore, the use of sibrotuzumab, a clinically validated antibody, supports the feasibility of FAP-targeted NIR-PIT in clinical applications. Further studies are needed to elucidate the detailed mechanisms underlying drug delivery improvement and to explore the potential of combining this therapy with other drugs to maximize therapeutic efficacy.

## Methods

### Sex as a biological variable.

Sex was not considered a biological variable in this study. All mice used were female to prevent unintended breeding, and no sex-based comparisons were performed. There was no specific scientific rationale for selecting females; however, as these mechanisms are not known to be sex dependent, the findings should apply to both sexes.

### Cell lines and culture methods.

This study used human cell lines from esophageal squamous cell carcinoma (TE4 and TE8), esophageal adenocarcinoma (OE19), pancreatic carcinoma (Panc-1 and MiaPaCa-2), fetal esophageal fibroblast 3 (FEF3), GFP-FEF3, and WI-38. Previous studies have reported on the expression of FEF3 and GFP-FEF3 (41).

TE4 and TE8 cells were purchased from the RIKEN BRC Cell Bank (Tsukuba, Japan), OE19 cells from the European Collection of Authenticated Cell Cultures (Salisbury, United Kingdom), Panc-1 and MiaPaCa-2 cells from the American Type Culture Collection, and WI-38 cells from the Health Science Research Resources Bank (Osaka, Japan).

TE4 and TE8 cells were cultured in RPMI 1640 supplemented with 10% FBS (Thermo Fisher Scientific) and 100 IU/mL penicillin and streptomycin (Sigma-Aldrich). WI-38 was cultured in MEM (Sigma-Aldrich) with the same supplements, while the remaining cell lines thrived in DMEM (Sigma-Aldrich), under conditions of 37°C and 5% CO_2_.

### Reagents.

Abraxane (100 mg for i.v. infusion) was obtained from Taiho Pharmaceutical Co. Vectibex (100 mg of panitumumab for i.v. infusion) was purchased from Takeda Pharmaceutical Co. The reagents were prepared in PBS for further use.

### Fibroblast activation by cancer cell supernatant.

Following the methodology described in a previous study (30), the fibroblasts were activated using conditioned medium (CM), which was the supernatant from cancer cells. To prepare CM (CM/TE4 and CM/TE8), we adhered to the protocol described in a previous study (30). Briefly, CM consisted of DMEM supplemented with 2% FBS, which was incubated with each cancer cell line for 72 hours. Fibroblasts were cultured in CM for 72 hours, after which they were defined as CAFs.

### Synthesis of IR700-conjugated sibrotuzumab or isotype, IR800-conjugated Abraxane or panitumumab, Alexa Fluor 568–conjugated panitumumab, and DIG-conjugated Abraxane, panitumumab, or isotype.

Sibrotuzumab (1 mg; TAB-211, Creative Biolabs) and the isotype control (02-7102, Thermo Fisher Scientific) were mixed with a 5-fold molar excess of IRDye700DX *N*-hydroxysuccinimide ester (IR700; LICOR Biosciences), dissolved in DMSO, and added to 0.3 mol/L Na_2_HPO_4_. The mixture was vortexed and incubated at room temperature for 2 hours before purification on a Sephadex G50 column (PD-10, GE Healthcare).

Similarly, Abraxane (1 mg of albumin, containing 0.25 mg of paclitaxel), panitumumab (1 mg), and the isotype control (02-7102, Thermo Fisher Scientific) were conjugated with a 5-fold molar excess of either IRDye800CW *N*-hydroxysuccinimide ester (IR800; LICOR Biosciences), Alexa Fluor 568 *N*-hydroxysuccinimide ester (Alexa568; A20003, Thermo Fisher Scientific), or digoxigenin (DIG), following the same procedure as for IR700. The conjugation reaction proceeded for 1 hour at room temperature.

The resulting antibody-photoabsorber conjugates were designated Sib-IR700, IgG-IR700, Abra-IR800, Pan-IR800, Pan-Alexa568, Abra-DIG, and Pan-DIG.

### Quality control for Sib-IR700.

We performed SDS-PAGE as quality control for Sib-IR700, as previously reported (56). Diluted sibrotuzumab was used as a non-conjugated control for SDS-PAGE, and fluorescent bands were measured using an IVIS Spectrum (PerkinElmer). An excitation filter (675 nm) and an emission filter (720 nm) were used for IR700.

### Clinical samples.

We enrolled 149 patients who underwent esophagectomy at the Department of Gastroenterological Surgery, Okayama University Hospital. The exclusion criteria were as follows: patients who had undergone endoscopic mucosal resection or endoscopic submucosal dissection before surgery, those diagnosed with cancer in situ (Tis, T1a) or M1 melanoma, or those who had received neoadjuvant therapy.

Ultimately, 84 samples were eligible for further analysis. We analyzed all patient data for age, sex, histological type, neoadjuvant therapy, pathological invasion depth (pT), and lymph node status (pN) using the TNM Classification of Malignant Tumors, 8th edition (Union for International Cancer Control) for tumor classification.

### IHC protocol for αSMA, FAP, Col I, HA, CD31, EGFR, and cytokeratin in clinical specimens, spheroids, and in vivo tumor specimens.

Paraffin-embedded tissue sections were deparaffinized and treated with 0.3% hydrogen peroxide (H_2_O_2_) in methanol for 10 minutes to block endogenous peroxidase activity. Antigen retrieval involved heating the sections in retrieval solution in a microwave oven for 14 minutes. After cooling to room temperature, sections were incubated with primary antibodies: anti-αSMA (A5228, Sigma-Aldrich) (1:1,000), anti-FAP (ab207178, Abcam) (1:250), anti–collagen I (ab34710, Abcam) (1:150), anti-CD31 for clinical specimens (3528, Cell Signaling Technology [CST]) (1:500), anti-CD31 for in vivo tumors (77699, CST) (1:500), anti-EGFR antibody (4267, CST) (1:200), and anti-cytokeratin (IS053, Dako) (1:500), all diluted in Dako Antibody Diluent (S0809). After incubation, the sections were treated with secondary antibodies in Dako Diluent, developed with DAB Substrate/Chromogen buffer (Dako) and counterstained with hematoxylin. Finally, the sections were dehydrated and mounted on a cover glass.

Biotinylated hyaluronan-binding protein (HABP; 385911, EMD Millipore Corp.) was used for HA detection. After antigen retrieval, the sections were blocked with an Avidin/Biotin Blocking Kit (415041, Nichirei Biosciences) and incubated with HABP (1:200) in Dako Diluent for 1 hour. Horseradish peroxidase–conjugated streptavidin (426062, Nichirei Biosciences) was applied, following the same visualization process as described above.

### Masson’s trichrome staining of in vivo tumor specimens.

Collagen detection was performed using Masson’s Trichrome Stain Kit (TRM-1, ScyTek Laboratories) per the manufacturer’s instructions.

### ICC protocol for Col I and HA in in vitro cells.

The cells were fixed and permeabilized with methanol for 30 minutes and blocked using Blocking One (03953-95, Nacalai Tesque Inc.) for 30 minutes. For Col I staining, the cells were incubated with an anti–Col I antibody (1:100) in Blocking One for 1 hour, followed by incubation with the secondary antibody (Alexa Fluor 568, A21069, Thermo Fisher Scientific) (1:100) in Blocking One for 30 minutes. Cells were stained with 0.1 μg/mL DAPI for 10 minutes.

For HA detection, cells were blocked with avidin and biotin after methanol fixation and Blocking One treatment. The cells were then incubated with HABP (1:200) in Blocking One for 1 hour, followed by incubation with horseradish peroxidase–conjugated streptavidin. For visualization, cells were treated with Opal 570 Reagent Pack (FP1488001KT, Akoya Biosciences Inc.) (1:100) in 1× Plus Amplification Diluent (FP1498A, Akoya Biosciences Inc.) for 10 minutes and stained with 0.1 μg/mL DAPI for 10 minutes.

The entire procedure was performed on ice, starting with fixation. Stained cells were observed using a BZ-X700 All-in-One Fluorescence Microscope (KEYENCE) with DAPI and Texas red filters.

### Flow cytometry analysis of FAP, sibrotuzumab, Col I, and EGFR in vitro.

FEF3 cells were seeded in 12-well plates, and the following day, the medium was replaced with DMEM containing 2% FBS or CM from TE4, TE8, OE19, Panc-1, or MiaPaCa-2 cells. After 72 hours of culturing, the cells were designated as NFs or CAFs. The cells were harvested using a cell scraper into PBS containing 2% FBS, centrifuged at 400*g* for 5 minutes at 4°C, and subsequently stained for specific targets.

For FAP and sibrotuzumab, the cells were incubated with PE-conjugated anti-FAP antibody (FAB3715P, R&D Systems) (1:50), PE-conjugated isotype control (IC002P, R&D Systems) (1:50), or Sib-IR700 conjugate (10 μg/mL) for 30 minutes. After staining, the cells were fixed with 4% paraformaldehyde (PFA) for 30 minutes and analyzed using a FACSLyric flow cytometer (BD Biosciences Inc.), with the PE and APC-Cy7 channels.

For Col I analysis, the cells were fixed with 4% PFA for 30 minutes, permeabilized using Intracellular Staining Permeabilization Wash Buffer (BioLegend), and stained with anti–Col I antibody (14695-1-AP, Proteintech Group Inc.) (1:500) or isotype control (3900, CST) (1:100) for 30 minutes. The cells were then incubated with Alexa Fluor 647 (A21245, Thermo Fisher Scientific) (1:100) for 15 minutes. The stained cells were analyzed using the same flow cytometer described above.

For EGFR analysis, TE8 cells were plated in 12-well plates and stained with anti-EGFR antibody (4267, CST) (1:200) or isotype control (3900, CST) (1:200) for 30 minutes, followed by Alexa Fluor 488 (A11008, Thermo Fisher Scientific) (1:100) for 15 minutes. The stained cells were analyzed using a FACSLyric flow cytometer, with the GFP channel used for Alexa Fluor 488 detection.

### ELISA protocol for HA measurement.

FEF3 cells were seeded in 12-well plates, and the following day, the medium was replaced with DMEM supplemented with 2% FBS or CM from TE4, TE8, OE19, Panc-1, or MiaPaCa-2 cells. After 72 hours of culture, the cells were categorized as NFs or CAFs, and the supernatant was collected for analysis.

The QnE Hyaluronic Acid ELISA Assay Kit (BTP-96200, Biotech Trading Partners) was used per the manufacturer’s instructions.

### Fluorescent imaging with propidium iodide after FAP-targeted NIR-PIT in vitro.

FEF3, GFP-FEF3, and WI-38 cells were seeded in a 12-well plate, and the supernatant was replaced with DMEM containing 2% FBS or CM from TE8 cells. After culturing for 3 days, the cells were incubated with either IgG-IR700 or Sib-IR700 (1 μg/mL) for 1 hour. After incubation, FEF3 cells incubated with IgG-IR700 or Sib-IR700 were observed using a BZ-X700 All-in-One Fluorescence Microscope with a Cy7 filter. Subsequently, the cells were irradiated with 690 nm laser light (BrixX695-2500UHP, Omicron-Laserage Laserprodukte GmbH) at an energy of 50 J/cm^2^. Energy was measured using an optical power meter (PM 100, Thorlabs Inc.). After irradiation, the cells were incubated with propidium iodide (PI; Sigma-Aldrich) (1:2,000). The treated cells were observed using a BZ-X700 All-in-One Fluorescence Microscope with a TRITC filter.

### Spheroid formation.

Using 96-well low-attachment plates (174925, Thermo Fisher Scientific), spheroids were formed with either TE8 alone or TE8 plus FEF3 to establish CAF-poor or CAF-rich models, respectively. The cell-seeding density was adjusted according to the specific requirements of each experiment.

### Spheroids for IHC analysis without FAP-targeted NIR-PIT.

TE8 cells (1 × 10^4^), with or without an equal number of FEF3 cells, were seeded onto plates and cultured in DMEM supplemented with 10% FBS for 2 days. Spheroids were then treated with Pan-DIG (10 μg/mL) for 6 hours, fixed with 4% PFA for 30 minutes, embedded in paraffin, and sectioned for analysis.

### Spheroids for IHC analysis after FAP-targeted NIR-PIT.

Spheroids were prepared using TE8 cells (1 × 10^4^ or 5 × 10^4^), with or without an equal number of FEF3 cells, and cultured in DMEM supplemented with 10% FBS for 1 day. They were then treated with Sib-IR700 (10 μg/mL) for 1 day, followed by NIR light irradiation at 50 J/cm^2^. At 1 hour or 2 days after irradiation, the spheroids were treated with either Pan-DIG and Abra-DIG for 1 hour or Pan-DIG for 6 hours, fixed with 4% PFA for 30 minutes, embedded in paraffin, and sectioned for subsequent analyses.

### 3D imaging protocol for spheroids.

TE8 cells were pre-stained with CellTracker Blue (2 μL/mL) for 30 minutes in a humidified incubator at 37°C and 5% CO_2_ before seeding. TE8 (1 × 10^4^) plus GFP-FEF3 (1 × 10^4^) cells were seeded into a 96-well low-attachment plate and cultured in DMEM supplemented with 10% FBS for 2 days to form spheroids.

The formed spheroids were fixed with 4% PFA for 30 minutes, followed by dehydration with ethanol. Tissue clearing was performed using a tissue-clearing reagent (5732, Corning).

Cleared spheroids were imaged using a Confocal Laser Scanning Microscope LSM780 (Carl Zeiss) in the *Z*-stack mode to capture detailed 3D structures.

The 3D images were reconstructed using IMARIS software.

### Fluorescent IHC for αSMA, Col I, digoxigenin, and HA on spheroid and in vivo tumor specimens.

Paraffin-embedded tissue sections were deparaffinized and blocked using Protein Block Serum-Free (X0909, Dako) for 5 minutes. Antigen retrieval was achieved by microwaving the sections in the retrieval solution for 14 minutes and allowing them to cool to room temperature.

Sections were incubated with primary antibodies: anti-αSMA (1:1,000), anti–Col I (1:150), and anti-DIG (700772, Thermo Fisher Scientific) (1:500), all diluted in Antibody Diluent.

After incubation with the primary antibody, the sections were treated with the following secondary antibodies: Alexa Fluor 488 (A11017, Thermo Fisher Scientific) (1:100) or Alexa Fluor 568 (A21069, Thermo Fisher Scientific), diluted in the same Antibody Diluent. For visualization, sections were mounted with NucBlue (P36981, Thermo Fisher Scientific) on a cover glass.

For hyaluronic acid staining, the sections were subjected to avidin-biotin blocking after antigen retrieval. They were then incubated with HABP (1:200) in Antibody Diluent for 1 hour, followed by a reaction with a horseradish peroxidase–conjugated Streptavidin Kit. Opal 570 Reagent Pack (1:100) in 1× Plus Amplification Diluent was used for 10 minutes for fluorescent labeling, and the sections were finally sealed with NucBlue on a cover glass.

### Flow cytometry protocol for Pan-IR800 on spheroids.

FEF3 cells were stained with CytoTell UltraGreen (22240, AAT Bioquest Inc.) for 30 minutes in a humidified incubator at 37°C with 5% CO_2_ to differentiate between TE8 and FEF3 in the CAF-rich model.

TE8 (1 × 10^4^) or TE8 (1 × 10^4^) plus FEF3 (1 × 10^4^) cells were seeded into a 96-well low-attachment plate and cultured in DMEM supplemented with 10% FBS for 2 days. The spheroids were treated with Pan-IR800 (1 μg/mL) for 6 hours. Four spheroids were then transferred into individual Eppendorf tubes and centrifuged at 400*g* for 5 minutes at 4°C, and the supernatant was discarded. The spheroids were digested with collagenase II (300 μg) and dispase (600 μg) in 300 μL of PBS.

After digestion, 700 μL of PBS with 2% FBS was added, followed by another centrifugation at 400*g* for 5 minutes at 4°C. The cells were fixed in 4% PFA for 10 minutes. Flow cytometric analysis was performed using a FACSLyric instrument employing GFP and APC-Cy7 channels. This experiment was performed in triplicate.

### Flow cytometry protocol for αSMA and Pan-Alexa568 on spheroids after FAP-targeted NIR-PIT.

TE8 (5 × 10^4^) or TE8 (5 × 10^4^) plus FEF3 (5 × 10^4^) cells were seeded in 96-well low-attachment plates and cultured in DMEM supplemented with 10% FBS for 1 day. The spheroids were treated with Sib-IR700 (10 μg/mL) for 1 day, followed by NIR light irradiation at a dose of 50 J/cm^2^. Two days after irradiation, the spheroids were incubated with Pan-Alexa568 for 6 hours.

Two spheroids were then transferred to each Eppendorf tube and centrifuged at 400*g* for 5 minutes at 4°C, and the supernatant was discarded. Spheroids were digested with a mixture of collagenase II (300 μg) and dispase (600 μg) in 300 μL PBS. After addition of 700 μL of PBS with 2% FBS and another centrifugation step at 400*g* for 5 minutes at 4°C, cells were fixed with 4% PFA for 10 minutes. Permeabilization was performed with Intracellular Staining Permeabilization Wash Buffer, followed by incubation with anti-αSMA antibody (1:250) for 30 minutes, and then with Alexa Fluor 488 (A11017, Thermo Fisher Scientific) (1:100) for 15 minutes.

Flow cytometry analysis was conducted using a FACSLyric system with appropriate fluorescence compensation settings. The GFP channel was used for Alexa Fluor 488 detection, and the PE channel for Alexa568.

### Animal study protocol.

Athymic female BALB/c-nu/nu mice, aged 4–5 weeks, were procured from Clea and used for experiments at 6–8 weeks of age.

We used only a bilateral subcutaneous model. TE8 cells (2 × 10^6^) for the CAF-poor mouse model or TE8 (2 × 10^6^) plus FEF3 (6 × 10^6^) cells for the CAF-rich mouse model were suspended in PBS (50 μL) plus Matrigel Matrix (50 μL) (356234, Corning) and inoculated into both flanks subcutaneously.

Daily monitoring was conducted, and tumor size and body weight were measured twice weekly. Tumor volume (mm^3^) was calculated using the following formula: length × width^2^ × 0.5. Mice were euthanized when the tumors reached the humane endpoint.

The mice were housed in a pathogen-free environment at the Okayama University Animal Laboratory.

### Imaging panitumumab after FAP-targeted NIR-PIT.

In the CAF-rich model, Sib-IR700 (50 μg) was administered intraperitoneally at tumor sizes of 100 or 500 mm^3^. Only the treatment side (right side) of the mouse was irradiated with NIR light; the opposite side was covered with aluminum foil 1 day after administration. Pan-IR800 (100 μg) or Pan-DIG (100 μg) was administered i.v. 1 hour after irradiation. For Pan-IR800, the mice were imaged using the IVIS Spectrum. An excitation filter (675 nm) and an emission filter (720 nm) were used for IR700. Excitation filter 745 and emission filter 800 were used for IR800. For Pan-DIG, the mice were sacrificed 1 hour after irradiation.

### Imaging Abraxane with IVIS Spectrum after FAP-targeted NIR-PIT.

In the CAF-rich model, Sib-IR700 (100 μg) was administered intraperitoneally at a tumor size of 100 mm^3^. Only the treatment side (right side) of the mouse was irradiated with NIR light; the opposite side was covered with aluminum foil 1 day after administration. Abra-IR800 (100 μg) or Abra-DIG (100 μg) was administered i.v. 1 hour after irradiation. For the Abra-IR800, IVIS Spectrum imaging was conducted using the corresponding filters for IR700 and IR800. For Abra-DIG, the mice were sacrificed 1 hour after irradiation.

### In vivo tumor volume with Abraxane.

In both CAF-poor and CAF-rich models, Abraxane (400 μg) was administered i.v. in only the treatment group at a tumor size of 100 mm^3^. Euthanasia was performed at a tumor volume of 1,500 mm^3^ by CO_2_ inhalation.

### In vivo tumor volume with FAP-targeted NIR-PIT and Abraxane.

In the CAF-rich model, Sib-IR700 (100 μg) or IgG-IR700 (100 μg) was intraperitoneally administered at a tumor size of 100 mm^3^. One day after administration, only the treatment side (right side) of the mouse was irradiated with NIR light; the opposite side was covered with aluminum foil 1 day after administration. One hour after irradiation, Abraxane (400 μg) was administered intravenously. The mice were euthanized at a tumor volume of 1,000 mm^3^ with CO_2_ inhalation.

### Image analysis.

To select the regions for analysis, 3 intratumoral areas considered representative were identified on each slide. Within these regions, the areas with the highest expression levels were chosen for quantification. Their mean values were then calculated. Similarly, for CD31-positive microvasculature, the 3 representative regions with the highest density were selected per slide to measure the maximum minor axis of each vessel. This process was repeated 3 times for each sample. For clinical samples, all tissue sections were examined under ×200 magnification following established methods for FAP (32) and αSMA (14). For the in vitro and in vivo experiments, images were selected at ×100 magnification.

IHC analysis was performed using ImageJ software (NIH; https://imagej.net/ij/). For fluorescent immunostaining, a BZ-X Analyzer (KEYENCE) was used to ensure accurate fluorescence detection and analysis.

### Statistics.

Overall survival was evaluated using the Kaplan-Meier method, and differences between curves were assessed using the log-rank test. Spearman’s correlation analysis was used to assess the association between variables. For categorical data, proportions were compared using Fisher’s exact test. Group comparisons were conducted using the Mann-Whitney *U* test, unpaired 2-tailed *t* test, or paired 2-tailed *t* test, as appropriate. For analyses involving multiple groups, analysis of variance (ANOVA) followed by Tukey’s test was used. Statistical analyses were performed using GraphPad Prism version 10 (RRID: GPA-2796073; GraphPad Software). Statistical significance was set at *P* < 0.05.

### Study approval.

This study was conducted in accordance with the Declaration of Helsinki’s ethical standards and the Ethical Guidelines for Medical and Health Research Involving Human Subjects issued by the Ministry of Health, Labour and Welfare and the Ministry of Education, Culture, Sports, Science and Technology of Japan. The use of these clinical samples was approved by the Ethics Review Board of Okayama University, Okayama, Japan (approval 1801-023). The study was conducted using an opt-out procedure approved by the Ethics Review Board of Okayama University (protocol no. 1801-023), and the requirement for written informed consent was waived. The experimental animal protocol was approved and reviewed by the Ethics Review Committee for Animal Experiments at Okayama University (approval OKU-2021550 and OKU-2021845).

### Data availability.

The datasets used and analyzed during the current study are available in the Supporting Data Values XLS file.

## Author contributions

SN, KN, TO, SK, HT, YS, H Kawai, and TF contributed to the concept and study design. SN and KN contributed to the development of the methodology. SN, TM, YT, TT, HM, H Kawai, KK, MA, ST, T Kunitomo, T Kobayashi, and H Kashima contributed to data acquisition (e.g., provided animals, acquired and managed patients, provided facilities). SN and KN contributed to the analysis and interpretation of data (e.g., statistical analysis, biostatistics, and computational analysis). SN, KN, T Kato, NN, PLC, and TF contributed to the writing, reviewing, and/or revising the manuscript. SN, KN, YS, HT, and TF provided to the administrative, technical, or material support (e.g., reporting or organizing data, constructing databases). KN, HT, YS, H Kobayashi, and TF contributed to the study supervision.

## Funding support

This work is the result of NIH funding, in whole or in part, and is subject to the NIH Public Access Policy. Through acceptance of this federal funding, the NIH has been given a right to make the work publicly available in PubMed Central.

- Japan Society for the Promotion of Science KAKENHI (grant 20K09009).
- Intramural Research Program of the National Institutes of Health, National Cancer Institute, Center for Cancer Research (grant ZIA BC 011513 to H Kobayashi ).

## Supplementary Material

Supplemental data

Unedited blot and gel images

Supplemental video 1

Supplemental video 2

Supplemental video 3

Supplemental video 4

Supporting data values

## Version 1 12/22/2025

Electronic publication
