# Supplementary material for: Enhancement of drug delivery through fibroblast activation protein–targeted near-infrared photoimmunotherapy
Source: JCI Insight. 2025 Dec 22;10(24):e195776. doi: 10.1172/jci.insight.195776 (PMC12890525; doi:10.1172/jci.insight.195776)
Supplement: Supplemental data [file jciinsight-10-195776-s109.pdf]

Supplemental Information for

Enhancement of drug delivery through fibroblast activation protein-targeted near-infrared photoimmunotherapy

Seitaro Nishimura[1], Kazuhiro Noma[1]*, Tasuku Matsumoto[1], Yasushige Takeda[1],Tatsuya Takahashi[1], Hijiri Matsumoto[1], Kento Kawasaki[1], Hotaka Kawai[2], Tomoyoshi Kunitomo[1], Masaaki Akai[1], Teruki Kobayashi[1], Noriyuki Nishiwaki[1], Hajime Kashima[1], Takuya Kato[1], Satoru Kikuchi[1], Shunsuke Tanabe[1], Toshiaki Ohara[13], Hiroshi Tazawa[14], Yasuhiro Shirakawa[15], Peter L Choyke[6], Hisataka Kobayashi[6], and Toshiyoshi Fujiwara[1]

[1] Department of Gastroenterological Surgery, Okayama University Graduate School of Medicine, Dentistry, and Pharmaceutical Science, Okayama, Japan.

[2] Department of Oral Pathology and Medicine, Okayama University Graduate School of Medicine, Dentistry, and Pharmaceutical Science, Okayama, Japan.

[3] Department of Pathology and Experimental Medicine, Okayama University Graduate School of Medicine, Dentistry, and Pharmaceutical Science, Okayama, Japan.

[4] Center for Innovative Clinical Medicine, Okayama University Hospital, Okayama, Japan.

[5] Department of Gastroenterological Surgery, Hiroshima City Hiroshima Citizens Hospital, Hiroshima, Japan.

[6] Molecular Imaging Branch, Center for Cancer Research, National Cancer Institute, National Institutes of Health, Bethesda, MD, USA.

Correspondence: Dr. Kazuhiro Noma, Department of Gastroenterological Surgery, Okayama University Graduate School of Medicine, Dentistry, and Pharmaceutical Sciences, 2-5-1 Shikata-cho, Kita-ku, Okayama 700-8558, Japan. Phone: +81-86-235-7255; Fax: +81-86-221-8775; E-mail: knoma@md.okayama-u.ac.jp.

List of Supplemental Information

Supplemental Figure 1: Patient enrollment and analysis workflow for esophageal cancer

Supplemental Figure 2: Relationship between ECM components, FAP, αSMA, and vascular morphology in esophageal Cancer

Supplemental Figure 3: In vitro evaluation of Col I, HA, and FAP expression in CAFs and NFs

Supplemental Figure 4: Analysis of ECM, EGFR expression, and drug penetration in CAF-poor and CAF-rich spheroid models

Supplemental Figure 5: Validation of Sib-IR700 and NIR-PIT-induced cytotoxicity in WI38 (control) and WI38 (TE8) cells

Supplemental Figure 6: Evaluation of FAP-targeted NIR-PIT effects on CAF-rich spheroids: Cell viability, αSMA reduction, and Col I reduction

Supplemental Figure 7: Impact of FAP-targeted NIR-PIT on drug delivery and fluorescent intensity in CAF-rich tumor models

Supplemental Figure 8: Evaluation of FAP-targeted NIR-PIT effects on Sib-IR700 and Abra-IR800 distribution in CAF-rich tumor models

Supplemental Figure 9: Differential effects of Abraxane on tumor growth in CAF-poor and CAF-rich tumor models

Supplemental Figure 10: Tumor growth, weight, and body weight analysis in IgG-IR700 and Sib-IR700-treated mouse models with or without NIR light

Supplemental Video Legends (Video 1-4)

Supplemental Table 1: Clinicopathological features for collagen I in esophageal cancer patients

Supplemental Table 2: Clinicopathological features for hyaluronic acid and 0–9 μm vessel in esophageal cancer patients

A

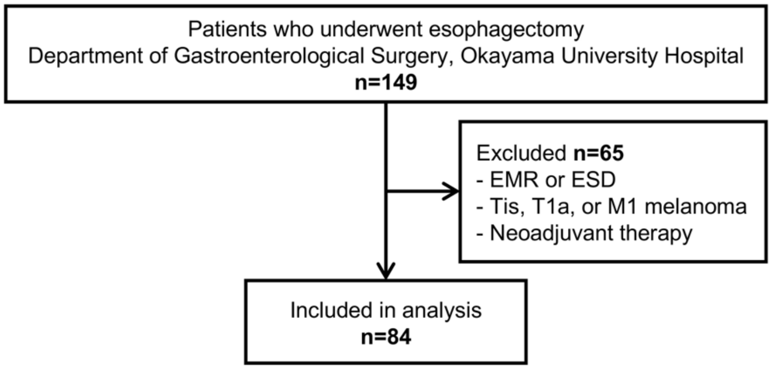

Supplemental Figure 1. Patient enrollment and analysis workflow for esophageal cancer

A. Patient enrollment flowchart

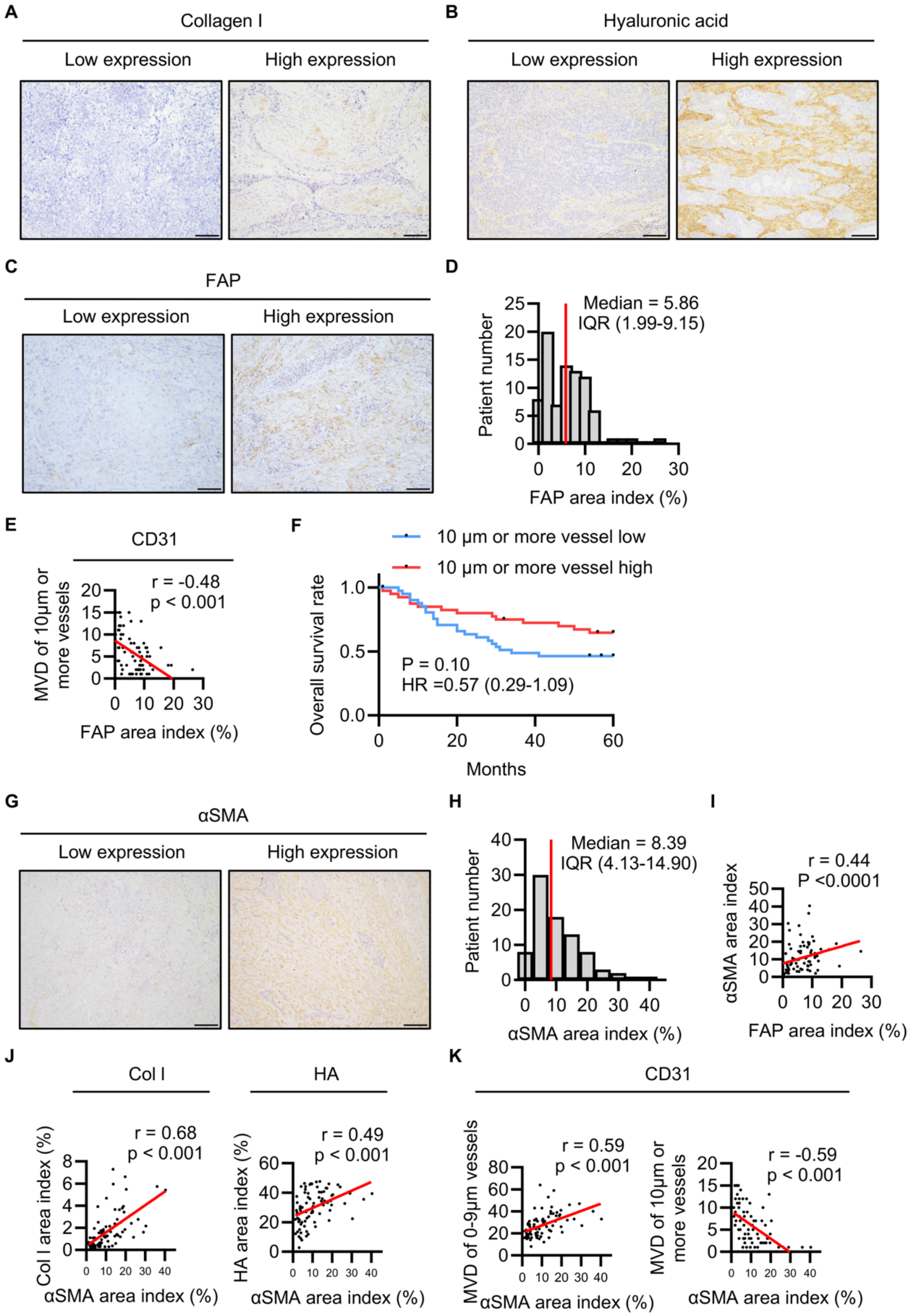
A
Collagen I
Low expression
High expression
B
Hyaluronic acid
Low expression
High expression
C
FAP
Low expression
High expression
D
Median = 5.86
IQR (1.99-9.15)
Patient number
FAP area index (%)
E
CD31
r = -0.48
p < 0.001
MVD of 10μm or more vessels
FAP area index (%)
F
10 μm or more vessel low
10 μm or more vessel high
P = 0.10
HR =0.57 (0.29-1.09)
Overall survival rate
Months
G
αSMA
Low expression
High expression
H
Median = 8.39
IQR (4.13-14.90)
Patient number
αSMA area index (%)
I
r = 0.44
P <0.0001
αSMA area index
FAP area index (%)
J
Col I
r = 0.68
p < 0.001
Col I area index (%)
αSMA area index (%)
HA
r = 0.49
p < 0.001
HA area index (%)
αSMA area index (%)
K
CD31
r = 0.59
p < 0.001
MVD of 0-9μm vessels
αSMA area index (%)
r = -0.59
p < 0.001
MVD of 10μm or more vessels
αSMA area index (%)

Supplemental Figure 2. Relationship between ECM components, FAP, αSMA, and vascular morphology in esophageal cancer

A. Representative esophageal cancer sample showing Col I staining (200× magnification, scale bar: 100 μm). **B.** Representative esophageal cancer sample showing HA staining (200× magnification, scale bar: 100 μm). **C.** Representative esophageal cancer sample showing FAP staining (200× magnification, scale bar: 100 μm). **D.** Histogram showing FAP area indices, with the median marked (red bar). **E.** Correlation between FAP levels and MVD of vessels ≥10 μm in diameter in esophageal cancer samples (n = 84, Spearman's correlation). **F.** Survival comparison for patients with high (n = 45) and low (n = 39) MVD of 10 μm or more diameter vessels (log-rank test). **G.** Representative esophageal cancer sample showing αSMA staining (200× magnification, scale bar: 100 μm). **H.** Histogram showing αSMA area indices, with the median marked (red bar). **I.** Correlation between FAP and αSMA area indices in esophageal cancer samples (n = 84, Spearman's correlation). **J.** Correlation between αSMA levels and Col I/HA area indices in esophageal cancer samples (n = 84, Spearman's correlation). **K.** Correlation between αSMA levels and MVD of 0–9 μm diameter vessels and 10 μm or more diameter vessels in esophageal cancer samples (n = 84, Spearman's correlation).

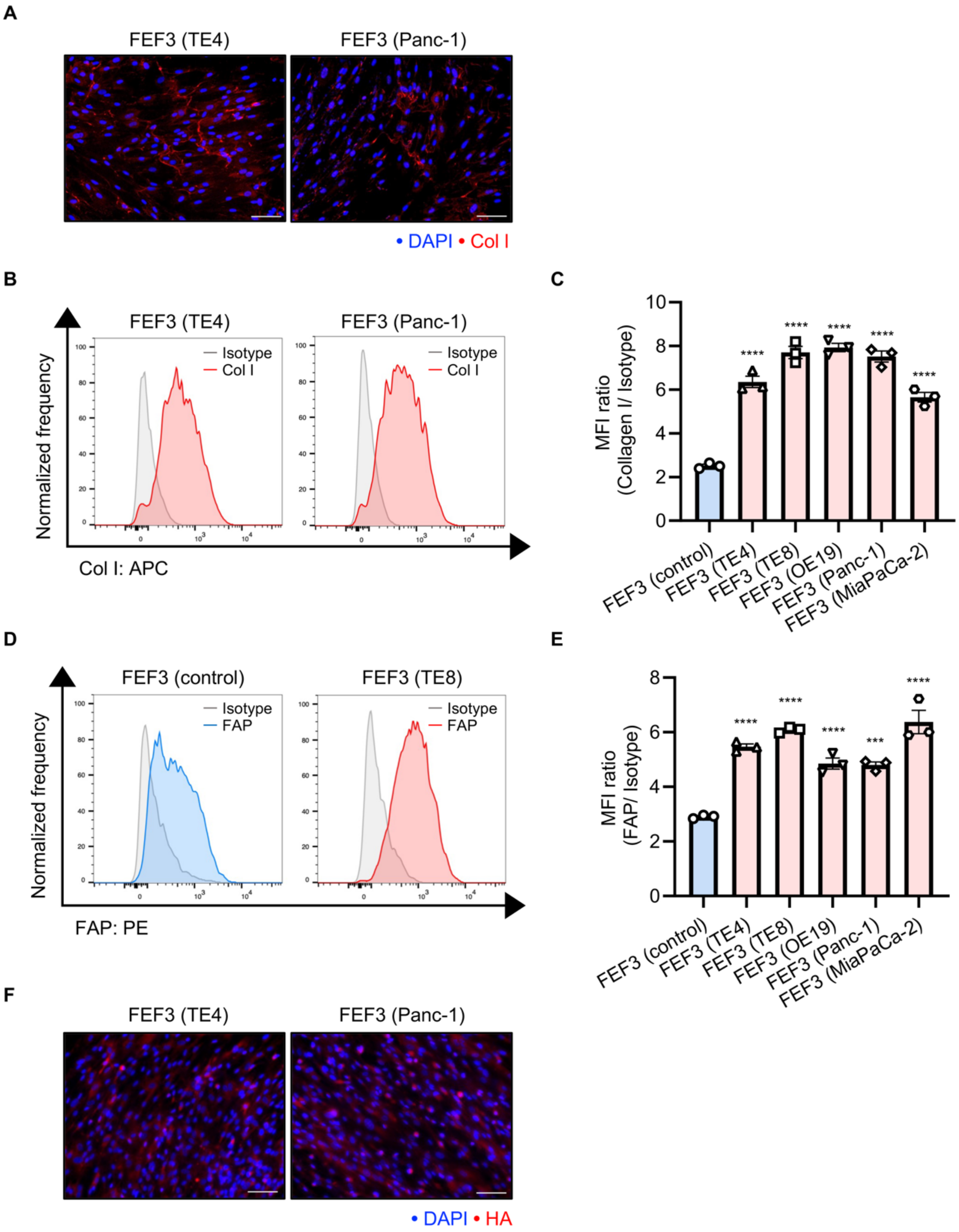
A
FEF3 (TE4)
FEF3 (Panc-1)
• DAPI • Col I
B
FEF3 (TE4)
FEF3 (Panc-1)
Isotype
Col I
Normalized frequency
Col I: APC
C
MFI ratio (Collagen I/ Isotype)
****
FEF3 (control)
FEF3 (TE4)
FEF3 (TE8)
FEF3 (OE19)
FEF3 (Panc-1)
FEF3 (MiaPaCa-2)
D
FEF3 (control)
FEF3 (TE8)
Isotype
FAP
Normalized frequency
FAP: PE
E
MFI ratio (FAP/ Isotype)
****
***
FEF3 (control)
FEF3 (TE4)
FEF3 (TE8)
FEF3 (OE19)
FEF3 (Panc-1)
FEF3 (MiaPaCa-2)
F
FEF3 (TE4)
FEF3 (Panc-1)
• DAPI • HA

Supplemental Figure 3. In vitro evaluation of Col I, HA, and FAP expression in CAFs and NFs

A. ICC images comparing Col I expression in FEF3 (TE4 and Panc-1), Col I (red) and DAPI (blue), at 200× magnification (scale bar: 100 μm). **B.** Histogram showing Col I expression levels in FEF3 (control) and FEF3 (TE4 and Panc-1). **C.** Comparative analysis of Col I expression between FEF3 (control) and FEF3 (TE4, TE8, OE19, Panc-1 and MiaPaCa-2) in FCM (n = 3 per group; mean ± SEM; one-way ANOVA with Tukey's test). **D.** Histogram showing FAP expression levels in FEF3 (control) and FEF3 (TE8). **E.** Comparative analysis of FAP expression between FEF3 (control) and FEF3 (TE4, TE8, OE19, Panc-1 and MiaPaCa-2) in FCM (n = 3 per group; mean ± SEM; one-way ANOVA with Tukey's test). **F.** ICC images comparing HA expression in FEF3 (TE4 and Panc-1), Col I (red) and DAPI (blue), at 200× magnification (scale bar: 100 μm). Significance indicated as *, $P < .05$; **, $P < .01$; ***, $P < .001$; ****, $P < .0001$; N.S. (not significant).

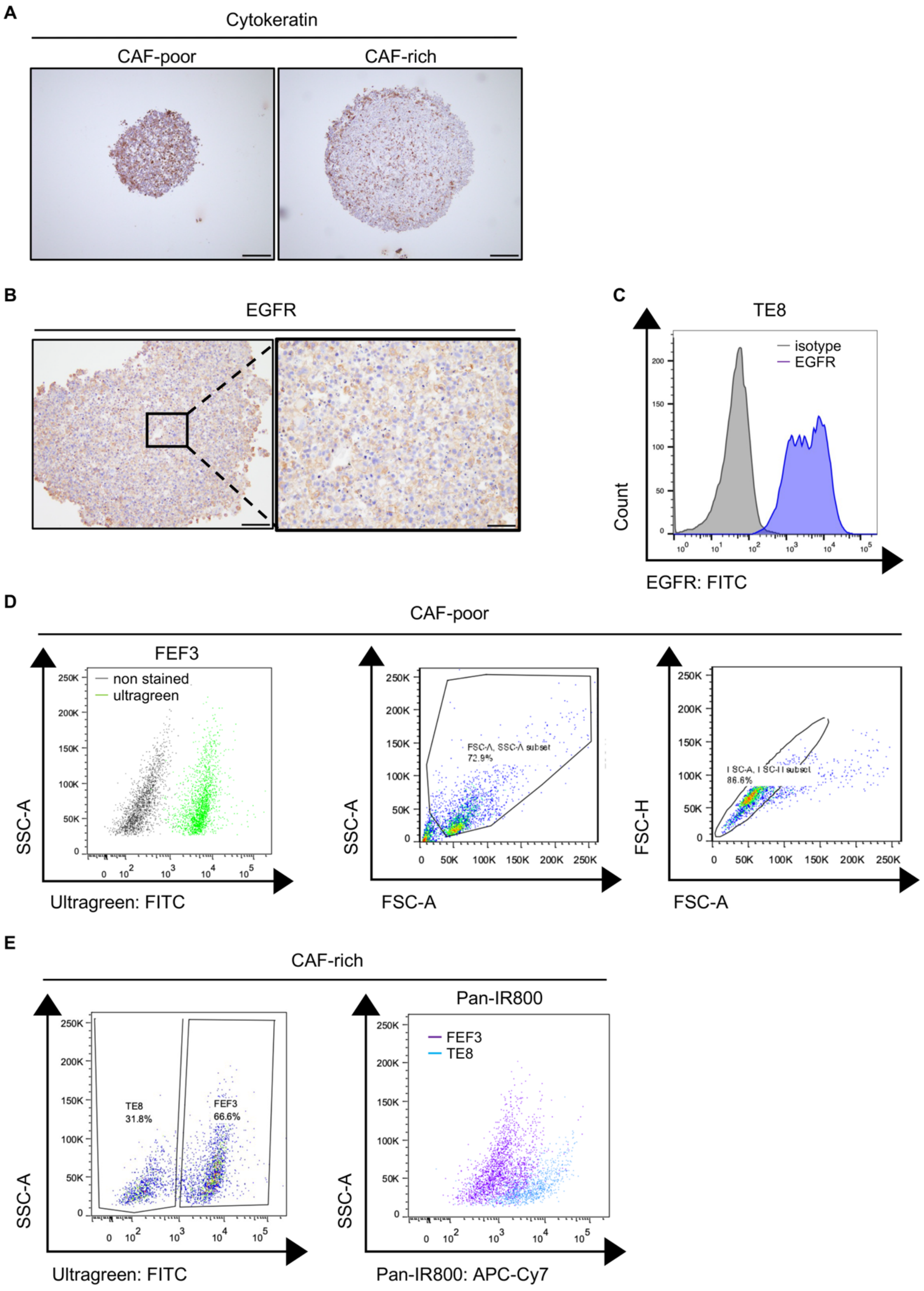
A
Cytokeratin
CAF-poor
CAF-rich
B
EGFR
C
TE8
isotype
EGFR
Count
EGFR: FITC
D
CAF-poor
FEF3
non stained
ultragreen
SSC-A
Ultragreen: FITC
FSC-A, SSC-A subset
72.9%
SSC-A
FSC-A
FSC-H
FSC-A
E
CAF-rich
TE8
31.8%
FEF3
66.6%
SSC-A
Ultragreen: FITC
Pan-IR800
FEF3
TE8
SSC-A
Pan-IR800: APC-Cy7

Supplemental Figure 4. Analysis of ECM, EGFR expression, and drug penetration in CAF-poor and CAF-rich spheroid models

CAF-poor spheroids (human TE8 cells only) and CAF-rich spheroids (human TE8 cells co-cultured with human FEF3 cells at a 1:1 ratio) were used throughout this figure.

A. IHC staining for cytokeratin in CAF-poor and CAF-rich spheroids at 100× magnification (scale bar: 200 μm). **B.** IHC staining for EGFR in CAF-poor spheroids at 200× magnification (right, scale bar: 100 μm) and 400× magnification (left, scale bar: 50 μm). **C.** Histogram of EGFR binding in TE8 cells. **D.** Flow cytometry of CAF-poor spheroids: Left: Ultragreen fluorescence intensity in FEF3 cells, distinguishing between non-stained (black) and ultragreen-stained populations (green). Middle: FSC-A and SSC-A gating, identifying 72.9% of the gated population as live cells. Right: FSC-A and FSC-H gating identifying 86.9% of the gated population for doublet discrimination. **E**. Flow cytometry analysis of CAF-rich spheroids. Left: Ultragreen fluorescence intensity compared to TE8 cells (unstained) and FEF3 cells (stained with ultragreen). Right: Pan-IR800 fluorescence intensity in FEF3 (blue) and TE8 (purple) cells.

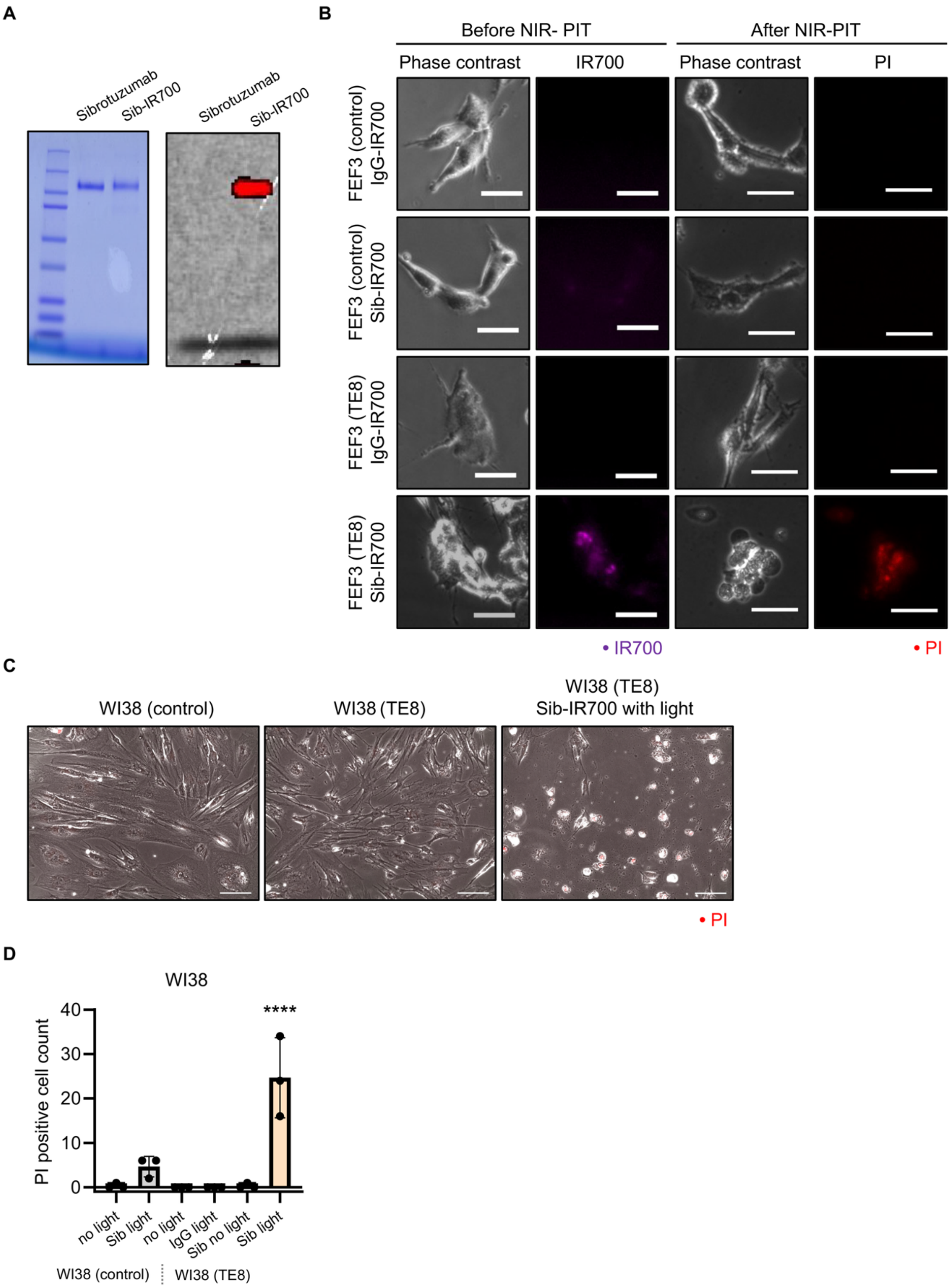

A
Sibrotuzumab
Sib-IR700
Sibrotuzumab
Sib-IR700
B
Before NIR- PIT
After NIR-PIT
Phase contrast
IR700
Phase contrast
PI
FEF3 (control) IgG-IR700
FEF3 (control) Sib-IR700
FEF3 (TE8) IgG-IR700
FEF3 (TE8) Sib-IR700
• IR700
• PI
C
WI38 (control)
WI38 (TE8)
WI38 (TE8) Sib-IR700 with light
• PI
D
WI38
PI positive cell count
40
30
20
10
0
****
no light
Sib light
no light
IgG light
Sib no light
Sib light
WI38 (control)
WI38 (TE8)

Supplemental Figure 5. Validation of Sib-IR700 and NIR-PIT-induced cytotoxicity in WI38 (control) and WI38 (TE8) cells

A. Validation of Sib-IR700 by SDS-PAGE. **Left:** Colloidal Blue staining. **Right:** IVIS spectrum with excitation filter 675 and emission filter 720. **B.** Representative ICC before and after NIR-PIT in FEF3 (control) and FEF3 (TE8) treated with IgG-IR700 or Sib-IR700, showing IR700 (magenta) and PI (red) (scale bar: 50 μm). **C.** Fluorescent images merged with phase contrast showing PI staining in WI38 (control) and WI38 (TE8) cells, untreated or treated with Sib-IR700 and NIR light; PI (red), at 200× magnification (scale bar: 100 μm). **D.** Comparative analysis of PI-positive cell counts across groups: untreated, IgG-IR700, or Sib-IR700, with or without NIR light (n = 3 per group; mean ± SEM; one-way ANOVA with Tukey's test). Statistical significance: *, $P < .05$; **, $P < .01$; ***, $P < .001$; ****, $P < .0001$; N.S. = not significant.

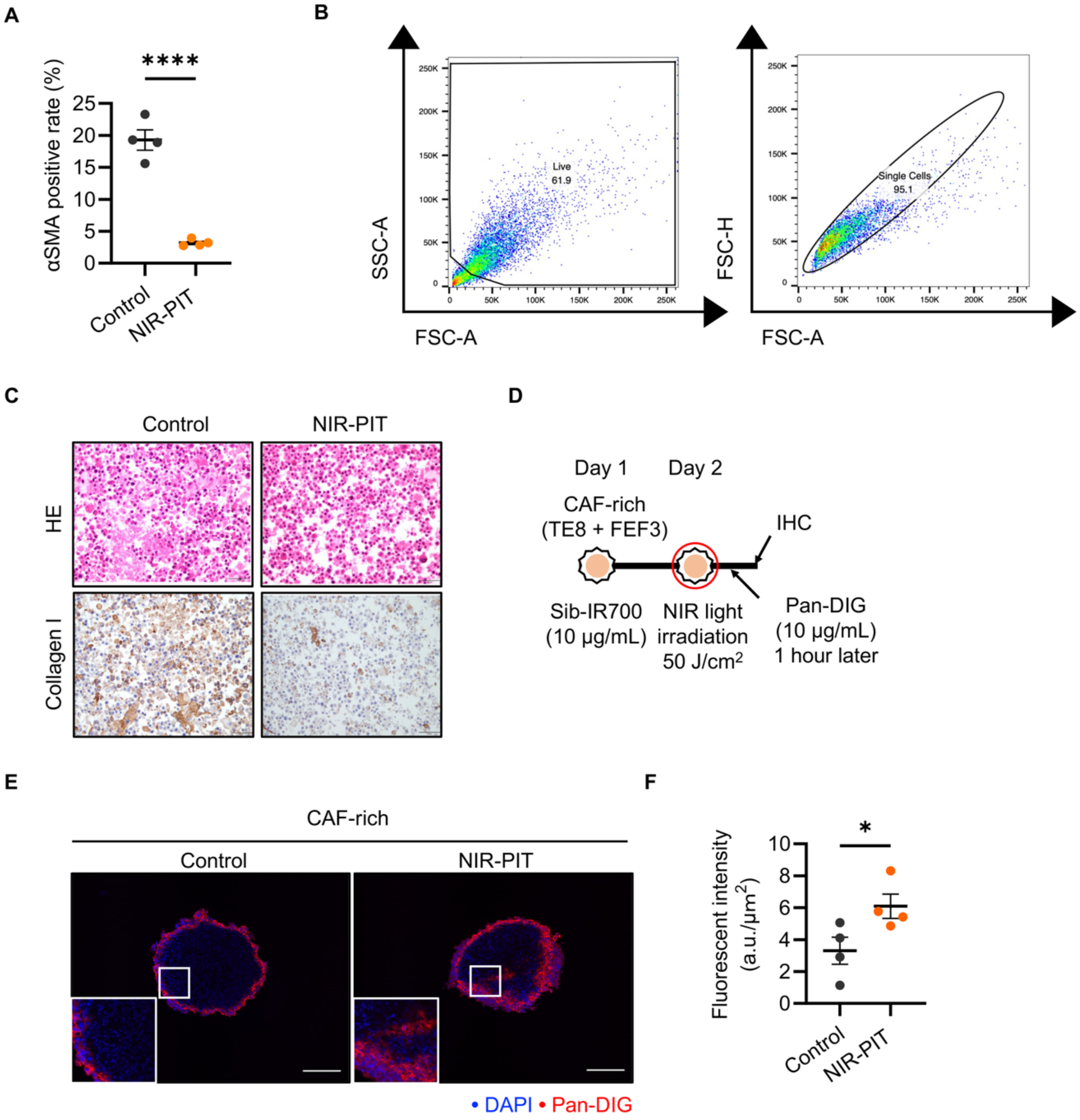
A
****
αSMA positive rate (%)
25
20
15
10
5
0
Control
NIR-PIT
B
SSC-A
FSC-A
Live
61.9
FSC-H
FSC-A
Single Cells
95.1
C
Control
NIR-PIT
HE
Collagen I
D
Day 1
Day 2
CAF-rich
(TE8 + FEF3)
IHC
Sib-IR700
(10 µg/mL)
NIR light
irradiation
50 J/cm2
Pan-DIG
(10 µg/mL)
1 hour later
E
CAF-rich
Control
NIR-PIT
• DAPI • Pan-DIG
F
*
Fluorescent intensity
(a.u./µm2)
10
8
6
4
2
0
Control
NIR-PIT

Supplemental Figure 6. Evaluation of FAP-targeted NIR-PIT effects on CAF-rich spheroids: Cell viability, αSMA reduction, and Col I reduction

CAF-rich spheroids (human TE8 cells co-cultured with human FEF3 cells at a 1:1 ratio) were used throughout this figure.

A. Quantitative analysis of αSMA-positive cells in CAF-rich spheroids, comparing control and NIR-PIT-treated groups 2 d post-treatment (n = 4; mean ± SEM; unpaired t-test). **B.** Flow cytometry analysis in CAF-rich spheroid models: Left: FSC-A and SSC-A gating, identifying 61.9% of the gated population as live cells. Right: FSC-A and FSC-H gating, identifying 95.1% of the gated population for doublet discrimination. **C.** HE and IHC staining for Col I in untreated CAF-rich control spheroids and NIR-PIT-treated CAF-rich spheroids 2 d post-treatment at 400× magnification (scale bar: 50 μm). **D.** Diagram of treatment schedule and NIR light irradiation for IHC in FAP-targeted NIR-PIT. **E.** Fluorescent IHC images showing Pan-DIG distribution in control and NIR-PIT-treated CAF-rich spheroids 1 h after treatment; DIG (red) and DAPI (blue), at 100× magnification (scale bar: 200 μm). **F.** Quantitative analysis of Pan-DIG fluorescence intensity per area (μm^2) in control and NIR-PIT-treated CAF-rich spheroids (n = 4; mean ± SEM; unpaired t-test). Statistical significance: *, P < .05; **, P < .01; ***, P < .001; ****, P < .0001; N.S. = not significant.

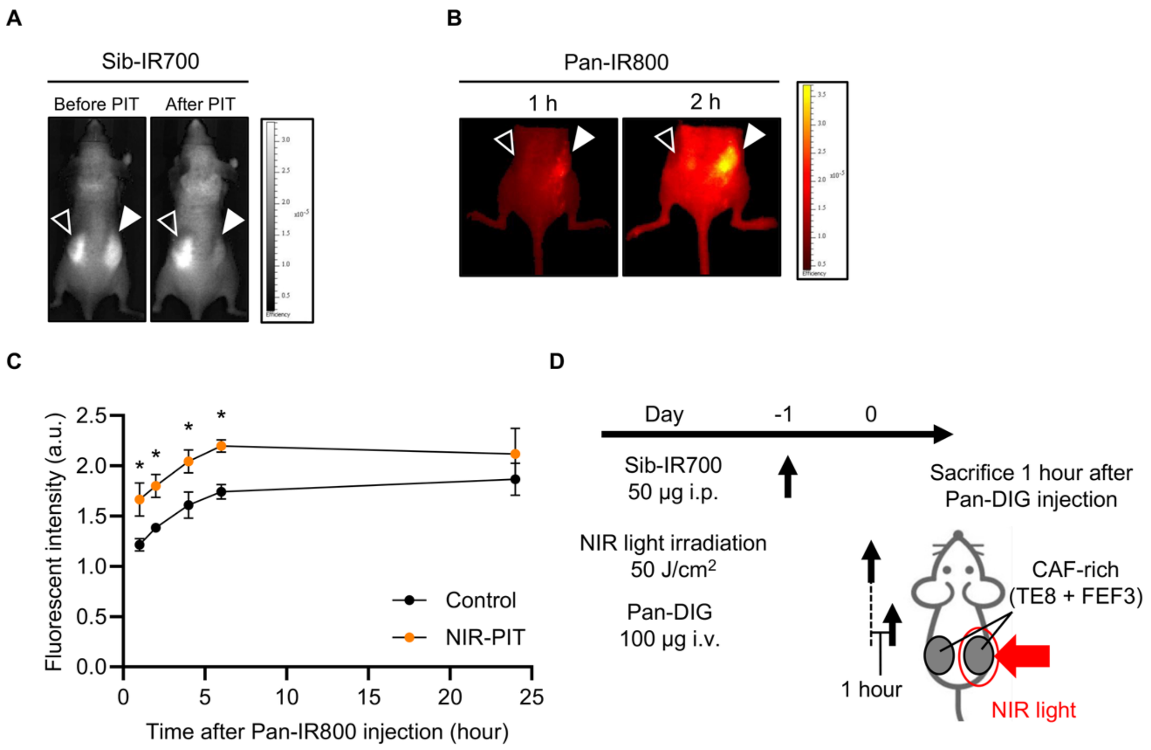

Supplemental Figure 7. Impact of FAP-targeted NIR-PIT on drug delivery and fluorescent intensity in CAF-rich tumor models

CAF-rich tumors (human TE8 cells co-cultured with human FEF3 cells at a 1:3 ratio) were used throughout this figure.

A. Sib-IR700 imaging before and after FAP-targeted NIR-PIT in a bilateral CAF-rich tumor model with tumors reaching 100 mm^3. Filled arrowheads indicate NIR-irradiated tumors (right), and open arrowheads indicate non-irradiated tumors (left). **B.** Pan-IR800 imaging in a bilateral CAF-rich tumor model following FAP-targeted NIR-PIT. Filled arrowheads show NIR-irradiated tumors (right), and open arrowheads show non-irradiated tumors (left). **C.** Fluorescent intensity ratio analysis (control/background vs. NIR-PIT-treated/background) over time post-Pan-IR800 administration (n = 4; mean ± SEM; repeated measures two-way ANOVA with Tukey's test). **D.** Schematic representation of the treatment protocol and NIR light exposure. Statistical significance: *, $P < .05$; **, $P < .01$; ***, $P < .001$; ****, $P < .0001$; N.S. = not significant.

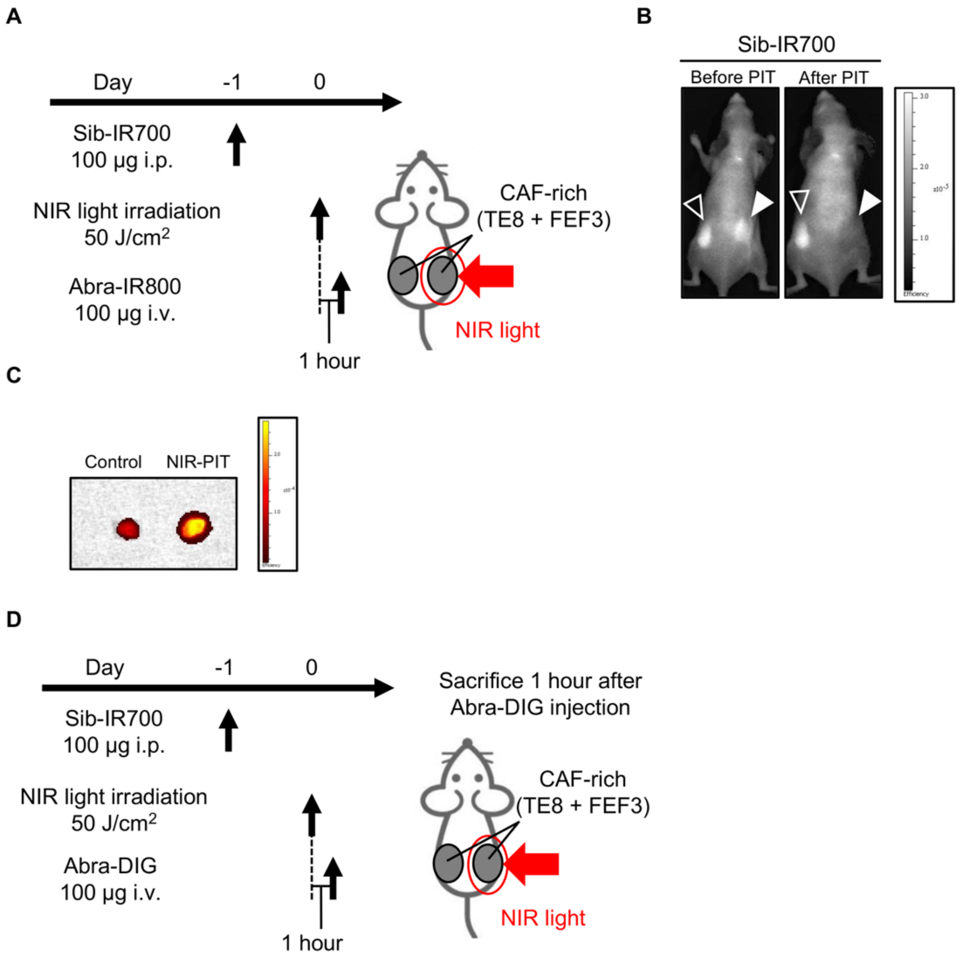

Supplemental Figure 8. Evaluation of FAP-targeted NIR-PIT effects on Sib-IR700 and Abra-IR800 distribution in CAF-rich tumor models

CAF-rich tumors (human TE8 cells co-cultured with human FEF3 cells at a 1:3 ratio) were used throughout this figure.

A. Diagram illustrating the treatment protocol and NIR light application. **B.** Sib-IR700 imaging captured before and after FAP-targeted NIR-PIT in a bilateral CAF-rich tumor model with tumors reaching 100 mm³. Filled arrowheads denote NIR-irradiated tumors (right), and open arrowheads represent non-irradiated tumors (left). **C.** Abra-IR800 imaging comparing untreated control tumors (left) to NIR-PIT-treated tumors (right) 1 h post-FAP-targeted NIR-PIT. **D.** Schematic outlining the treatment regimen and NIR light exposure setup.

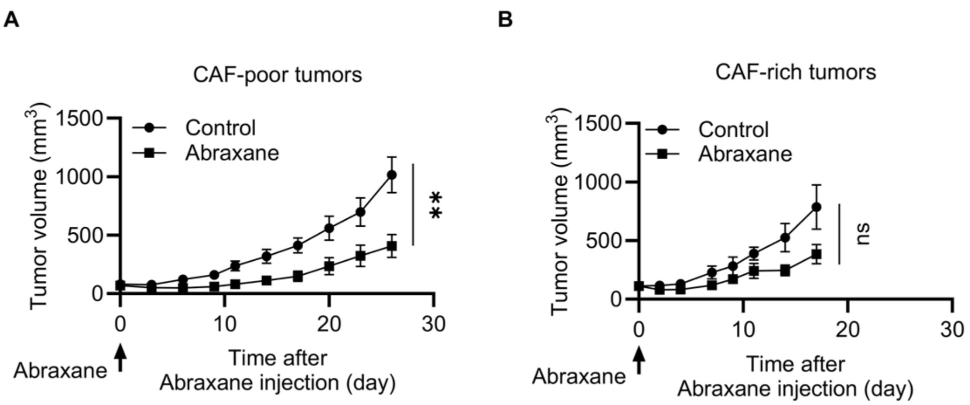

Supplemental Figure 9. Differential effects of Abraxane on tumor growth in CAF-poor and CAF-rich tumor models

CAF-poor tumors (human TE8 cells only) and CAF-rich tumors (human TE8 cells co-cultured with human FEF3 cells at a 1:3 ratio) were used throughout this figure.

A. Growth curve of bilateral CAF-poor tumors in mice, untreated (control) or treated with Abraxane (n = 4; mean ± SEM; one-way ANOVA with Tukey's test). **B**. Growth curve of bilateral CAF-rich tumors in mice, untreated (control) or treated with Abraxane (n = 3; mean ± SEM; one-way ANOVA with Tukey's test). Statistical significance: *, P < .05; **, P < .01; ***, P < .001; ****, P < .0001; N.S. = not significant.

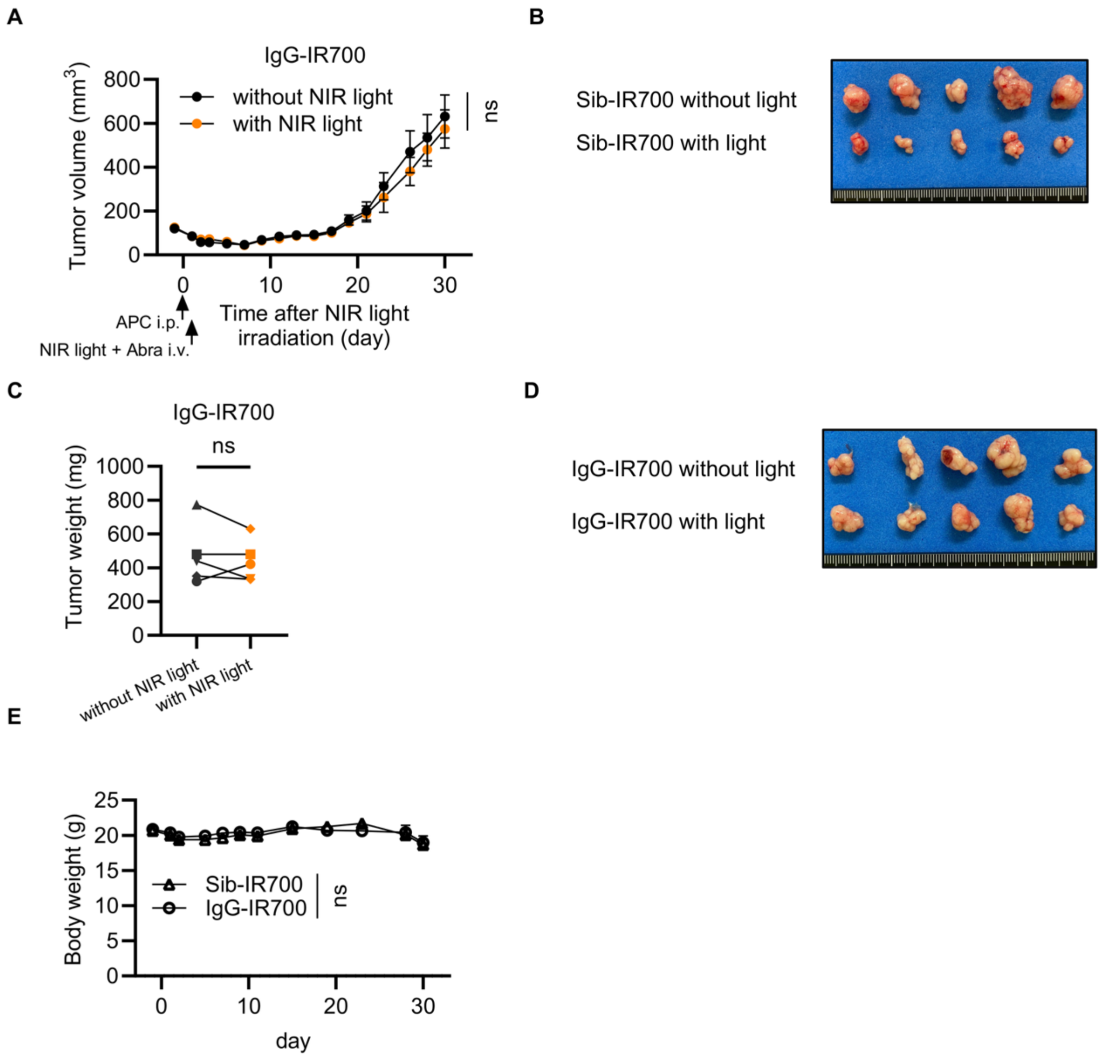

Supplemental Figure 10. Tumor growth, weight, and body weight analysis in IgG-IR700 and Sib-IR700-treated mouse models with or without NIR light

(CAF-rich) tumors (human TE8 cells co-cultured with human FEF3 cells at a 1:3 ratio) were used throughout this figure.

A. Tumor growth in mice injected with IgG-IR700, comparing groups with or without NIR light exposure (n = 5; mean ± SEM; one-way ANOVA with Tukey's test). **B.** Tumors excised from Sib-IR700-treated mice. The top row represents tumors without NIR light exposure, while the bottom row shows those exposed to NIR light. **C.** Tumor weight comparison between right-side

tumors exposed to NIR light and left-side tumors without light exposure in IgG-IR700-treated mice (n = 5; ratio paired t-test). **D.** Tumors retrieved from IgG-IR700-treated mice. The top row illustrates tumors without light exposure, and the bottom row displays tumors exposed to NIR light. **E.** Body weight analysis of mice treated with Sib-IR700 and IgG-IR700. Statistical significance: *, P < .05; **, P < .01; ***, P < .001; ****, P < .0001; N.S. = not significant.

Supplemental Video Legends

CAF-poor spheroids (human TE8 cells only) and CAF-rich spheroids (human TE8 cells co-cultured with human GFP-FEF3 cells at a 1:1 ratio) were used throughout these videos.

Supplemental Video 1. Original movie of spheroids showing CAF-poor spheroids labeled with DAPI (blue).

Supplemental Video 2. Original movie of spheroids showing CAF-rich spheroids labeled with GFP-FEF3 (green) and DAPI (blue).

Supplemental Video 3. Original movie of CAF-rich spheroids showing untreated (control) labeled with GFP-FEF3 (green) and DAPI (blue).

Supplemental Video 4. Original movie of CAF-rich spheroids showing FAP-targeted NIR-PIT-treated spheroids labeled with GFP-FEF3 (green) and DAPI (blue).

Supplemental Table 1: Clinicopathological features for collagen I in esophageal cancer patients.

Variable	Total (n=84)	Collagen I Low (n=42)	Collagen I High (n=42)	P value
Age (years)				
Median (IQR)	68 (62-73)	67.00 (45.00-85.00)	69.00 (40.00-85.00)	0.582§
Sex				1†
Male	67 (79.8%)	34 (81.0%)	33 (78.6%)	
Female	17 (20.2%)	8 (19.0%)	9 (21.4%)	
Tumor location				0.122†
Cervical	6 (7.1%)	1 (2.4%)	5 (11.9%)	
Upper	12 (14.3%)	9 (21.4%)	3 (7.1%)	
Middle	39 (46.4%)	19 (45.2%)	20 (47.6%)	
Lower	16 (19.1%)	6 (14.3%)	10 (23.8%)	
Abdominal	11 (13.1%)	7 (16.7%)	4 (9.5%)	
Pathological T stage				<0.001†*
T1	38 (45.2%)	32 (76.2%)	6 (14.3%)	
T2	11 (13.1%)	4 (9.5%)	7 (16.7%)	
T3	32 (38.1%)	5 (11.9%)	27 (64.3%)	
T4	3 (3.6%)	1 (2.4%)	2 (4.8%)	
Pathological N stage				0.009†*
N0	40 (47.6%)	26 (61.9%)	14 (33.3%)	
N1	21 (25.0%)	11 (26.2%)	10 (23.8%)	
N2	15 (17.9%)	4 (9.5%)	11 (26.2%)	
N3	8 (9.5%)	1 (2.4%)	7 (16.7%)	
Pathological stage				<0.001†*
I	27 (32.1%)	21 (50.0%)	6 (14.3%)	
II	21 (25.0%)	13 (31.0%)	8 (19.0%)	
III	27 (32.1%)	8 (19.0%)	19 (45.2%)	
IV	9 (10.7%)	0 (0.0%)	9 (21.4%)	
Histrogical type				1†
Squamous cell carcinoma	75 (89.3%)	37 (88.1%)	38 (90.5%)	
Adenocarcinoma	7 (8.3%)	4 (9.5%)	3 (7.1%)	
Other	2 (2.4%)	1 (2.4%)	1 (2.4%)	
αSMA Area Index				<0.001§*
Median (IQR)	8.39 (4.18-14.73)	4.37 (3.07-7.10)	14.56 (10.02-18.79)	
FAP Area Index				<0.001§*
Median (IQR)	5.86 (2.05-9.00)	2.82 (1.42-7.11)	8.10 (5.41-9.39)	

§, Mann–Whitney *U* test; †, Fisher’s exact test; IQR, Interquartile range

Supplemental Table 2: Clinicopathological features for hyaluronic acid and 0–9 µm vessel in esophageal cancer patients.

	Hyarulonic acid			0-9 µm vessel		
Variable	Low (n=42)	High (n=42)	P value	Low (n=43)	High (n=41)	P value
Age (years)						
Median (IQR)	67.50 (40.00-85.00)	68.00 (48.00-85.00)	0.74§	66.00 (40.00-85.00)	70.00(48.00-85.00)	0.234§
Sex			0.101†			0.28†
Male	30 (71.4%)	37 (88.1%)		32 (74.4%)	35 (85.4%)	
Female	12 (28.6%)	5 (11.9%)		11 (25.6%)	6 (14.6%)	
Tumor location			0.371†			0.594†
Cervical	2 (4.8%)	4 (9.5%)		2 (4.7%)	4 (9.8%)	
Upper	8 (19.0%)	4 (9.5%)		8 (18.6%)	4 (9.8%)	
Middle	16 (38.1%)	23 (54.8%)		18 (41.9%)	21 (51.2%)	
Lower	9 (21.4%)	7 (16.7%)		8 (18.6%)	8 (19.5%)	
Abdominal	7 (16.7%)	4 (9.5%)		7 (16.3%)	4 (9.8%)	
Pathological T stage			<0.001†*			<0.001†*
T1	31 (73.8%)	7 (16.7%)		29 (67.4%)	9 (22.0%)	
T2	4 (9.5%)	7 (16.7%)		5 (11.6%)	6 (14.6%)	
T3	6 (14.3%)	26 (61.9%)		8 (18.6%)	24 (58.5%)	
T4	1 (2.4%)	2 (4.8%)		1 (2.3%)	2 (4.9%)	
Pathological N stage			0.082†			0.106†
N0	24 (57.1%)	16 (38.1%)		26 (60.5%)	14 (34.1%)	
N1	11 (26.2%)	10 (23.8%)		9 (20.9%)	12 (29.3%)	
N2	6 (14.3%)	9 (21.4%)		5 (11.6%)	10 (24.4%)	
N3	1 (2.4%)	7 (16.7%)		3 (7.0%)	5 (12.2%)	
Pathological stage			0.001†*			0.001†*
I	21 (50.0%)	6 (14.3%)		22 (51.2%)	5 (12.2%)	
II	11 (26.2%)	10 (23.8%)		9 (20.9%)	12 (29.3%)	
III	9 (21.4%)	18 (42.9%)		9 (20.9%)	18 (43.9%)	
IV	1 (2.4%)	8 (19.0%)		3 (7.0%)	6 (14.6%)	
Histrogical type			1†			0.403†
Squamous cell carcinoma	37 (88.1%)	38 (90.5%)		39 (90.7%)	36 (87.8%)	
Adenocarcinoma	4 (9.5%)	3 (7.1%)		4 (9.3%)	3 (7.3%)	
Other	1 (2.4%)	1 (2.4%)		0 (0.0%)	2 (4.9%)	
αSMA Area Index			<0.001§*			<0.001§*
Median (IQR)	4.66 (3.21-8.03)	14.14 (8.68-18.16)		5.87 (3.25-8.39)	14.54 (9.09-18.48)	
FAP Area Index			<0.001§*			0.006§*
Median (IQR)	2.42 (1.42-6.06)	8.48 (5.41-9.63)		4.00 (1.46-8.03)	7.85 (5.01-9.68)	

§, Mann–Whitney *U* test; †, Fisher's exact test; IQR, Interquartile range
