# Supplementary figures and images for: Enhancement of drug delivery through fibroblast activation protein–targeted near-infrared photoimmunotherapy
Source: JCI Insight. 2025 Dec 22;10(24):e195776. doi: 10.1172/jci.insight.195776 (PMC12890525; doi:10.1172/jci.insight.195776)

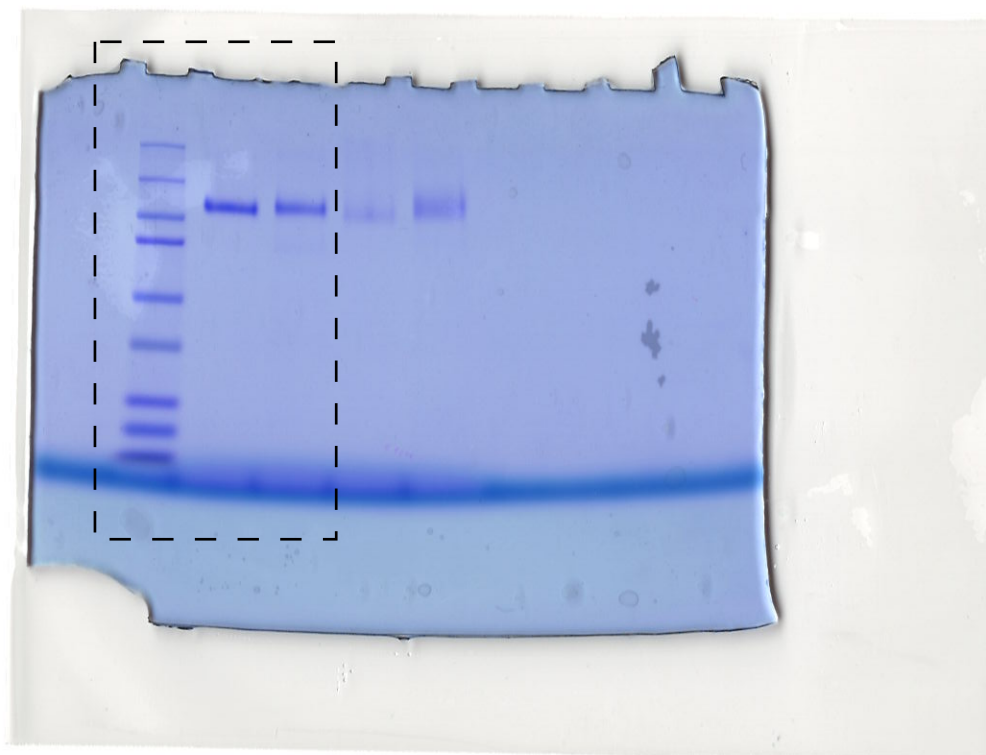

Supplement: Unedited blot and gel images [file jciinsight-10-195776-s110.pdf]
